# Prognostic role of miR-17-92 family in human cancers: evaluation of multiple prognostic outcomes

**DOI:** 10.18632/oncotarget.19096

**Published:** 2017-07-08

**Authors:** Feifei Liu, Feng Zhang, Xiangyu Li, Qi Liu, Wei Liu, Peng Song, Ziying Qiu, Yu Dong, Hao Xiang

**Affiliations:** ^1^ Department of Global Health, School of Health Sciences, Wuhan University, Wuhan, 430071, China; ^2^ Global Health Institute, Wuhan University, Wuhan, 430071, China

**Keywords:** miR-17-92 family, cancer, prognosis, meta-analysis

## Abstract

Recent evidence indicates that miR-17–92 family might be an essential prognostic biomarker for human cancers. However, results are still inconsistent. We therefore performed a meta-analysis to evaluate the predictive role of miR-17–92 family in human cancer prognosis. We searched literatures published before March 31th, 2017 inPubMed, Cochrane and Embase databases. Twenty six studies were included in our analyses. The overall hazard ratios (HRs) showed that high expression level of miR-17-92 family was a predictor of poor overall survival (OS): adjusted HRs = 1.71, 95% confidence intervals (CIs): 1.39–2.11, *p* < 0.00001, and poor disease-free survival (DFS): adjusted HRs = 2.29, 95% CIs: 1.41–3.72, *p* = 0.0008. However, no association between miR-17-92 family expression and cancer progress-free survival (PFS) was found (*p* > 0.05). Subgroup analyses showed that high expression of miR-17-92 family was associated with poor OS (adjusted HRs = 1.89, 95% CIs: 1.43–2.49, *p* < 0.00001) and DFS (adjusted HRs = 2.83, 95% CIs: 1.59–5.04, *p* = 0.0003) among the Asian, and no association was found for the Caucasian (*p* > 0.05). Besides, the HRs of miR-17-92 family high expression in tissue and serum samples was 1.68 (1.35–2.09) and 2.20 (1.08–4.46) for OS, and 1.73 (0.80–3.74) and 3.37 (2.25–5.02) for DFS. It also found that high expression of miR-17-92 family predicted a poor OS in breast cancer, esophageal squamous cell carcinoma, lymphoma and other cancers. Findings suggest that miR-17-92 family can be an effective predictor for prognosis prediction in cancer patients.

## INTRODUCTION

Cancer is now the second leading cause of death worldwide after cardiovascular disease, with cases of nearly all types of cancer on the rise [1]. GLOBOCAN reported 14.1 million new cancer diagnoses and 8.2 million deaths due to cancer in 2012 worldwide [2]. One reason for the high mortality of cancer is the lack of understanding of the molecular biology, which leads to the difficulty of identifying reliable biomarkers for disease detection and effective therapeutic agents in clinical applications [3]. Malignant tumors account for a large proportion of cancer cases, most of which have advanced local invasion and/or distant metastases at the time of diagnosis [4–6]. This leads to losing of operational opportunities for treatment. Therefore, identifying reliable biomarkers classify cancer patients into high- and low-risk groups for prognosis evaluation at an early stage and to improve treatment decision-making is important to lower the mortality. With the development of profiling studies, microRNAs (miRNAs) has been shown as one of the most promising biomarkers for the implications of cancer progression.

MiRNAs are small non-coding RNAs of approximately 20–22 nucleotides in length that play vital roles in the regulation of gene expression at the post-transcriptional level [7]. Alterations of miRNAs in the expression of oncogene (e.g. miR-17-92 family) or tumor suppressor (e.g. miR-34b and miR-520e) have been associated with carcinogenesis, malignant change, metastasis, and response to anti-cancer treatments [7–9]. Studies have shown that miRNAs are also potential prognostic factors in cancer development [10, 11], suggesting that miRNAs can be acting as prognostic biomarkers to guide treatment decisions in clinic. For instance, miR-17-92 family has been found as tumor promoters in different human cancers.

The miR-17-92 family maps to human chromosome 13 (13q31.3) and encodes for the miR-17–92 cluster (miR-17, miR-18a, miR-19a, miR-20a, miR-19b-1, miR-92a) and two paralogs (miR-106a, miR-106b) [12]. Several studies have shown that miR-17-92 family can be a potential prognostic marker in human cancers. Chen et al. [13] found that multiple myeloma patients with high expression of miR-17, miR-20, and miR-92 had shorter progression-free survival (PFS) compared to those with low expression. Similar results were also reported in other studies [14, 15]. However, some studies found the contrary role of miR-17-92 family in cancer prognosis. Sofie et al. [16] demonstrated that patients with high level of miR-92a had better clinical outcomes than patients with low level. Huang et al. [17] found that the over-expression of miR-19b was significantly correlated with longer disease-free survival (DFS) and overall survival (OS) in patients with hepatocellular carcinoma (HCC). Although inconsistent results have been reported, the miR-17-92 family consists of undeniably attractive members that play important roles in cancer prognosis.

Contradictory results in previous studies may due to small sample sizes and different detection methods. Further studies are needed to explore the relationship between miR-17-92 family expression and the prognosis of cancer patients. Evidence from previous studies indicate that clustered miRNAs with similar sequences may regulate a set of mRNA targets and therefore function as powerful regulators of specific cellular activities [18]. A cluster of miRNAs may be a better predictor of survival than a single miRNA [19]. We therefore performed this meta-analysis to evaluate the overall effect of the total eight miR-17-92 family members instead of a single miRNA.

## RESULTS

### Search results

**Figure 1:**
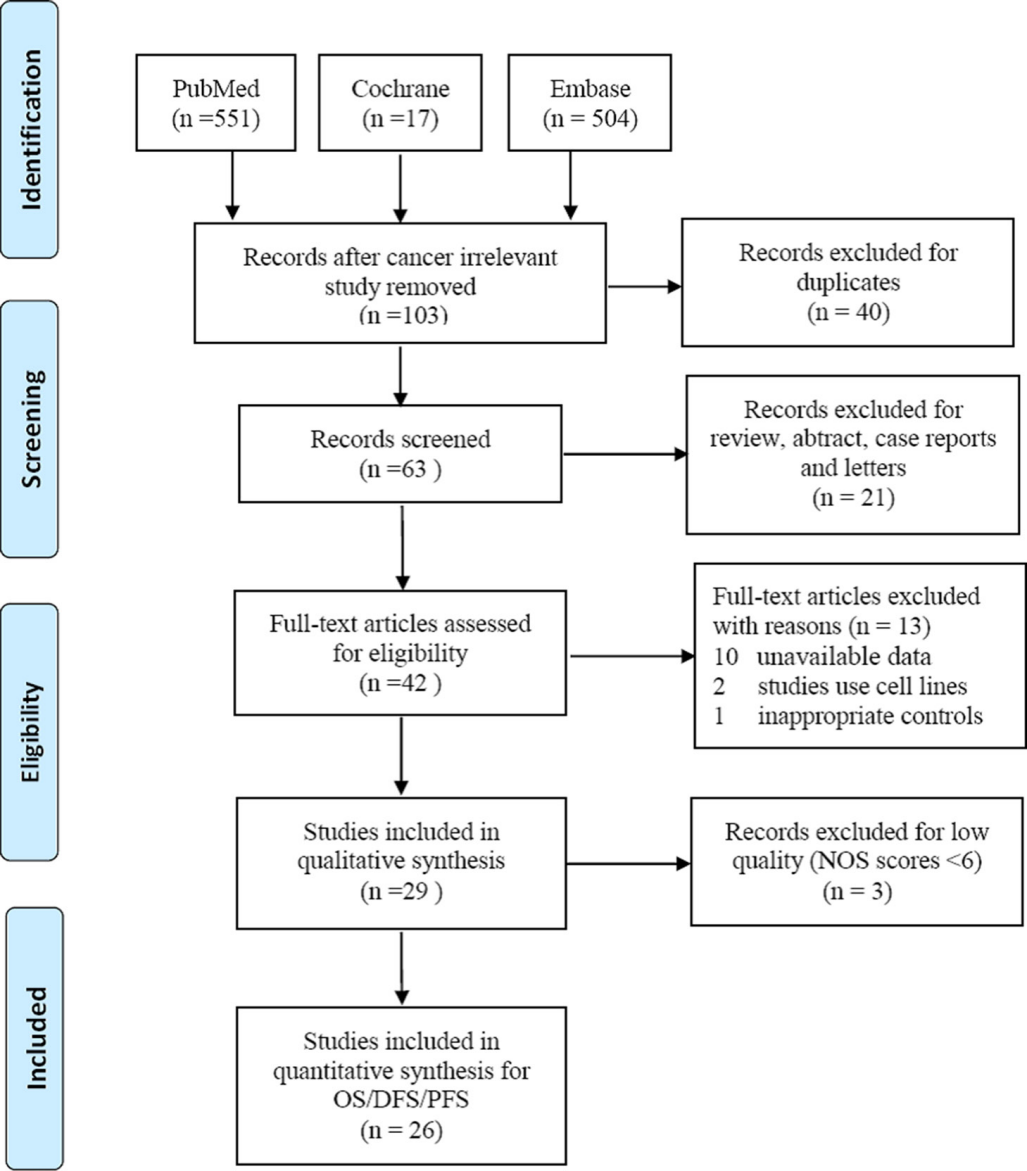
Flow chat of selecting studies for meta-analyses

A total of 1072 articles were retrieved from the PubMed, Cochrane, and Embase databases, of which 969 were excluded for irrelevant to cancers; 40 were duplicates; 21 were abstracts, review articles or letters; 10 articles did not contain available data; 2 studies used cell lines; and 1 study used inappropriate controls (did not use tumor tissues or serum samples). Finally, a total of 29 studies included in these analyses were assessed for quality using the Newcastle–Ottawa scale (NOS). Three studies with low quality (NOS scale less than 6) were then excluded. Eventually, 26 [15–17, 20–42] studies were included in this meta-analyses, with acceptable quality (scored between 6 and 8 in NOS). The selection process of studies for meta-analyses was shown in Figure 1.

The main characteristics of included studies included in the present analysis was summarized in Table 1 and Table 2. Of these 26 studies, 20 [15, 17, 21–30, 32–37, 39, 41] studies were conducted among the Asian, and the rest [16, 20, 31, 38, 40, 42] were among the Caucasian. Besides this, 21 [16, 17, 20–23, 27–29, 31–42] studies used tumor tissues to detect miRNAs concentrations, 4 [15, 24–26] studies used blood samples, and 1 [30] study tested in both tissue and serum samples. Among these research, 23 [15, 17, 20–22, 25–42] reported patient OS, 10 [16, 17, 20, 23–25, 28, 30, 36, 42] investigated DFS, and 3 [31, 32, 40] focused on PFS. Results were analyzed separately for studies reporting OS, DFS, or PFS simultaneously. We extract data from these 26 studies, which included 3189 participants in total. The type of cancers assessed in these studies included colorectal cancer (CRC) [20–25], lung cancer [15, 26, 27], hepatocellular carcinoma (HCC) [17, 28, 29], breast cancer (BC) [16, 30], esophageal squamous cell carcinoma (ESCC) [31, 32], glioma [33, 34], pancreatic cancer (PC) [35, 36], BL (including T-cell lymphoblastic lymphoma [37], Burkitt lymphoma [38]) and others (astrocytoma [39], gastrointestinal cancer [40], melanoma [41], and ependymomas [42]). Quantitative real-time PCR (qRT-PCR) was used in 25 studies to assess miRNA expression, and only 1 study exploited microarray analysis.

**Table 1:** Characteristic of studies included in the meta-analysis

First Author	Year	Country	Cancer Type	Sample Type	Assay Method	Number of patients	Median or Mean age	Average Follow-up (month)	NOS Scale			
									Selection	Comparability	Outcome	Total score
Raquel Diaz [20]	2008	Spain	CRC	Tissue	qRT-PCR	110	69.0	68	3	2	3	8
GE YU [21]	2011	China	CRC	Tissue	qRT-PCR	96	63.0	59.5	3	1	3	7
Tong Zhou [22]	2013	China	CRC	Tissue	RT-PCR	82	–	–	3	1	2	6
J-X Zhang [23]	2013	China	CRC	Tissue	qRT-PCR	138	–	66	3	1	3	7
Jialu Li -1 [24]	2015	China	CRC	Serum	RT-qPCR	85	58.7	36	3	1	2	6
Jialu Li -2 [24]	2015	China	CRC	Serum	RT-qPCR	90	56.6	32	3	1	2	6
T Matsumura [25]	2015	Japan	CRC	Serum	qRT-PCR	209	–	–	4	1	2	7
Qun Chen [26]	2013	China	Lung cancer	Serum	qRT-PCR	221	–	–	3	2	1	6
QUNYING LIN [15]	2013	China	NSCLC	Serum	qRT-PCR	201	58	23	3	2	3	8
Chaohui Wu [27]	2014	China	NSCLC	Tissue	qRT-PCR	61	–	–	3	2	1	6
Ming-Qi Fan [28]	2013	China	HCC	Tissue	qRT-PCR	100	–	–	3	2	1	6
Bin-Kui Li [29]	2014	China	HCC	Tissue	qRT-PCR	104	–	–	3	2	3	8
C-L Hung [17]	2015	China	HCC	Tissue	qRT-PCR	81	–	–	3	2	1	6
Sofie [16]	2012	Sweden	BC	Tissue	qRT-PCR	144	65	78	3	2	2	7
R-H Zheng-1 [30]	2015	China	BC	Tissue	qRT-PCR	173	53.7	–	3	2	2	7
R-H Zheng-2 [30]	2015	China	BC	Serum	qRT-PCR	173	53.7	–	3	2	2	7
Yuxin Hu [31]	2010	Germany	ESCC	Tissue	RT-PCR	158	–	–	3	1	2	6
Xiao-Ling Xu [32]	2014	China	ESCC	Tissue	qRT-PCR	105	63	34.5	3	2	3	8
Shengkui Lu [33]	2012	China	Glioma	Tissue	qRT-PCR	108	43	–	3	1	2	6
S-G Zhao [34]	2013	China	Glioma	Tissue	qRT-PCR	156	48	10	3	1	2	6
Jun Yu [35]	2010	Japan	PC	Tissue	qRT-PCR	80	65.5	–	3	2	2	7
Namkung [36]	2016	Korean	PC	Tissue	microarrays	104	64.9	–	3	1	2	6
Yanfeng Xi [37]	2015	China	T-LBL	Tissue	qRT-PCR	57	18	–	3	2	2	7
Robaina [38]	2016	Brazil	BL	Tissue	qRT-PCR	41	7.4	38.5	2	2	3	7
Feng Zhi [39]	2010	China	astrocytoma	Tissue	qRT-PCR	124	48.5	34.3	3	2	3	8
AYERBES [40]	2011	Spain	GI cancer	Tissue	qRT-PCR	38	62.5	153 week	2	2	2	6
N. Lin [41]	2015	China	melanoma	Tissue	qRT-PCR	97	–	–	3	2	1	6
Zakrzewska [42]	2016	Poland	ependymomas	Tissue	qRT-PCR	53	5	–	3	2	2	7

**Table 2:** Hazard ratios and 95% CIs of mir-17-92 family

First Author	miRNA	Survival	Univariate analysis			Multivariate analysis		
			HR	95% CI	*P* Value	HR	95% CI	*P* Value
Raquel Diaz [20]	Mir-17	OS	0.77	0.33–1.80	0.55	–	–	–
	Mir-106a	OS	0.49	0.24–0.99	0.04	0.52	0.26–1.07	0.07
GE YU [21]	Mir-17	OS	–	–	–	2.67	1.31–6.82	0.007
	Mir-18a	OS	–	–	–	1.68	0.33–3.43	0.435
	Mir-19a	OS	–	–	–	0.87	0.71–4.38	0.752
	Mir-19b	OS	–	–	–	1.52	1.09–2.11	0.367
	Mir-106a	OS	–	–	–	2.59	0.79–6.37	0.098
Tong Zhou [22]	Mir-92a	OS	2.947	1.49–5.813	0.002	2.342	1.072–5.115	0.033
T Matsumura [25]	Mir-19a	OS	4.15	1.90–10.9	0.0001	2.49	1.12–6.61	0.023
Qun Chen [26]	Mir-17	OS	1.767	1.039–3.005	0.035	–	–	–
QUNYING LIN [15]	Mir-19a	OS	3.042	2.082–4.444	< 0.001	1.438	1.007–2.052	0.046
Chaohui Wu [27]	Mir-19b	OS	3.591	1.564–8.246	0.003	3.466	1.389–8.650	0.008
Ming-Qi Fan [28]	Mir-20a	OS	4.483	2.769–9.572	0.009	4.937	2.221–9.503	0.022
Bin-Kui Li [29]	Mir-106b	OS	2.445	1.299–4.605	0.004	2.002	1.130–6.977	0.027
C-L Hung [17]	Mir-19b	OS	–	–	–	0.318	0.120–0.846	0.022
R-H Zheng-1 [30]	Mir-106b	OS	11.446	–	0.001	4.882	1.019–23.385	0.04
R-H Zheng-2 [30]	Mir-106b	OS	13.77	–	0.001	6.926	1.447–33.143	0.01
Yuxin Hu [31]	Mir-20	OS	1.17	0.65–2.12	0.61	0.69	0.26–4.31	0.47
Xiao-Ling Xu [32]	Mir-17a	OS	–	–	–	2.849	1.258–6.455	0.012
	Mir-18a	OS	–	–	–	2.151	0.990–4.675	0.053
	Mir-19a	OS	–	–	–	3.471	1.110–10.857	0.032
Shengkui Lu [33]	Mir-17	OS	6.2	1.3–18.6	0.001	5.1	0.8–15.9	0.008
S-G Zhao [34]	Mir-106a	OS	0.430	0.273–0.677	< 0.001	0.504	0.297–0.854	0.011
Jun Yu [35]	Mir-17	OS	1.8	1.0–3.1	0.003	0.9	0.4–1.7	0.1
Namkung [36]	Mir-106b	OS	–	–	–	3.81	0.76–19.23	0.102
Yanfeng Xi [37]	Mir-17	OS	–	–	–	4.225	1.249–14.293	0.003
	Mir-19	OS	–	–	–	2.179	1.068–4.440	0.032
Robaina [38]	Mir-17	OS	–	–	–	8.945	2.150–37.212	0.003
Feng Zhi [39]	Mir-106a	OS	1.716	0.985–2.991	0.057	1.629	0.899–2.954	0.108
AYERBES [40]	Mir-17	OS	1.065	0.999–1.102	0.052	2.62	1.55–4.49	< 0.001
	Mir-20a	OS	1.027	1.009–1.046	0.003	1.065	1.003–1.130	0.04
N. Lin [41]	Mir-106b	OS	–	–	–	2.09	1.11–10.26	0.02
Zakrzewska [42]	Mir-17	OS	2.93	1.07–8.01	0.036	3.26	0.96–11.04	0.057
	Mir-19a	OS	1.23	0.46–3.31	0.678	1.00	0.29–3.39	0.999
	Mir-106b	OS	1.46	0.54–3.97	0.455	0.94	0.23–3.76	0.927
Raquel Diaz [20]	Mir-17	DFS	0.89	0.38–2.09	0.78	–	–	–
	Mir-106a	DFS	0.49	0.23–0.95	0.03	0.35	0.16–0.76	0.009
J-X Zhang [23]	Mir-20a	DFS	0.47	0.22–1.03	0.058	–	–	–
	Mir-20a	DFS	0.59	0.30–1.13	0.11	–	–	–
	Mir-20a	DFS	0.54	0.36–0.80	0.112	–	–	–
	Mir-106b	DFS	0.42	0.16–1.07	0.072	–	–	–
	Mir-106b	DFS	0.46	0.19–1.11	0.083	–	–	–
	Mir-106b	DFS	0.49	0.32–0.74	0.0007	–	–	–
Jialu Li -1 [24]	Mir-17	DFS	3.72	1.61–8.60	0.002	3.74	1.34–10.40	0.012
	Mir-106a	DFS	4.31	1.02–18.27	0.03	3.34	1.29–8.62	0.013
Jialu Li -2 [24]	Mir-17	DFS	3.09	1.33–7.24	0.009	3.74	1.34–10.40	0.011
	Mir-106a	DFS	2.61	1.14–5.98	0.023	3.34	1.28–8.63	0.01
T Matsumura [25]	Mir-19a	DFS	4.15	1.90–10.9	0.0001	2.49	1.12–6.61	0.023
Ming-Qi Fan [28]	Mir-20a	DFS	4.591	2.933–8.457	0.015	4.281	3.316–6.741	0.013
C-L Hung [17]	Mir-19b	DFS	–	–	–	0.455	0.245–0.845	0.013
Sofie [16]	Mir-92a	DFS	0.382	0.138–0.781	0.012	0.375	0.145–0.972	0.043
R-H Zheng-1 [30]	Mir-106b	DFS	8.087	–	< 0.001	3.998	1.069–14.954	0.039
R-H Zheng-2 [30]	Mir-106b	DFS	10.457	–	< 0.001	5.561	1.487–20.803	0.01
Namkung [36]	Mir-106b	DFS	–	–	–	3.29	0.63–16.95	0.156
Zakrzewska [42]	Mir-17	DFS	4.78	1.79–12.76	0.002	4.96	1.67–14.68	0.004
	Mir-19a	DFS	1.47	0.63–3.42	0.373	1.47	0.51–4.25	0.784
	Mir-106b	DFS	1.31	0.56–3.06	0.527	0.89	0.26–3.01	0.855
Yuxin Hu [31]	Mir-20	PFS	1.09	0.60–1.98	0.77	0.66	0.24–1.81	0.42
Xiao-Ling Xu [32]	Mir-18a	PFS	–	–	–	1.832	1.044–3.165	0.040
	Mir-19a	PFS	–	–	–	3.317	1.032–10.650	0.045
AYERBES [40]	Mir-17	PFS	1.056	1.007–1.107	0.024	2.11	1.29–3.45	0.003
	Mir-20a	PFS	1.022	1.004–1.040	0.016	1.063	1.002–1.127	0.043

### MiR-17-92 family expression and cancer OS

A total of 23 studies were included for OS analyses, of which 15 [15, 20, 22, 25–29, 31, 33–35, 39, 40, 42] studies provided unadjusted OS, and 22 [15, 17, 20–22, 25, 27–42] studies provided adjusted values. We calculated the pooled HRs of OS separately.

In the unadjusted analyses among 15 studies, a significant heterogeneity was observed (*I*^2^ = 88%, *p* < 0.00001). In random effect model, results showed that higher expression level of miR-17-92 family was associated with poor OS (crude HRs = 1.56, 95% CIs: 1.31–1.86, *p* < 0.00001) (Supplementary Figure 1A). Subgroup analyses was ordinally conducted based on ethnicity, sample type, and cancer type. Results showed that high expression of miR-17-92 family was associated with poor OS among the Asian (crude HRs = 2.33, 95% CIs: 1.46–3.73, *p* = 0.0004), while no association was found for the Caucasian (Supplementary Figure 1B). Subgroup analyses by sample type, showed a significant association between high expression of miR-17-92 family and poor OS in both tissue (crude HRs = 1.36, 95% CIs: 1.14–1.61, *p* = 0.0005) and serum samples (crude HRs = 2.71, 95% CIs: 1.74–4.20, *p* < 0.00001) (Supplementary Figure 1C). Results of subgroup analyses by cancer type indicated that high expression of miR-17-92 family was an indicator of poor OS in lung cancer, HCC, and PC (*p* < 0.05) (Supplementary Figure 1D). (Table 3.)

**Table 3:** The pooled associations between mir-17-92 family and cancer prognosis

Sub group	OS						DFS						PFS					
	*N*	cHR 95% CI)	*P* value	*N*	aHR (95% CI)	*P* value	*N*	cHR (95% CI)	*P* value	*N*	aHR (95% CI)	*P* value	*N*	cHR (95% CI)	*P* value	*N*	aHR (95% CI)	*P* value
**Total**	15	1.56 (1.31–1.86)	< 0.00001	22	1.71 (1.39–2.11)	< 0.00001	7	1.22 (0.76–1.96)	0.41	9	2.29 (1.41–3.72)	0.0008	2	1.03 (1.01–1.04)	0.002	3	1.49 (0.95–2.34)	0.09
**Ethnic**																		
Asian	11	2.33 (1.46–3.73)	0.0004	16	1.91 (1.45–2.50)	< 0.00001	4	1.31 (0.70–2.44)	0.40	6	2.83 (1.59–5.04)	0.0004	–	–	–	1	2.05 (1.23–3.40)	0.006
Caucasian	4	1.04 (0.97–1.12)	0.23	5	1.37 (0.83–2.26)	0.22	3	1.06 (0.54–2.09)	0.86	3	1.48 (0.60–3.63)	0.39	2	1.03 (1.01–1.04)	0.002	2	1.23 (0.72–2.13)	0.45
**Sample Type**																		
Tissue	12	1.36 (1.14–1.61)	0.0005	20	1.68 (1.35–2.09)	< 0.00001	5	0.84 (0.50–1.42)	0.51	7	1.73 (0.80–3.74)	0.16	2	1.03 (1.01–1.04)	0.002	3	1.49 (0.95–2.34)	0.09
Serum	3	2.71 (1.74–4.20)	< 0.00001	3	2.20 (1.08–4.46)	0.03	2	3.43 (2.31–5.09)	< 0.00001	3	3.37 (2.25–5.02)	< 0.00001	–	–	–	–	–	–
**Cancer Type**																		
CRC	3	1.47 (0.53–4.08)	0.46	4	1.49 (0.98–2.27)	0.06	4	1.04 (0.64–1.68)	0.88	3	3.10 (2.15–4.48)	< 0.00001	–	–	–	–	–	–
Lung cancer	3	2.61 (1.75–3.89)	< 0.00001	2	2.01 (0.87–4.64)	0.10	–	–	–	–	–	–	–	–	–	–	–	–
HCC	2	3.43 (1.90–6.19)	< 0.0001	3	1.52 (0.39–5.83)	0.55	1	4.59 (2.93–7.19)	< 0.00001	2	1.42 (0.16–12.79)	0.75	–	–	–	–	–	–
BC	–	–	–	1	5.82 (1.92–17.60)	0.002	1	0.38 (0.14–1.06)	0.06	2	1.93 (0.31–11.87)	0.48	–	–	–	–	–	–
ESCC	1	1.17 (0.65–2.11)	0.60	2	1.96 (1.01–3.78)	0.05	–	–	–	–	–	–	–	–	–	–	–	–
Glioma	2	1.46 (0.11–19.84)	0.77	2	1.34 (0.14–12.63)	0.80	–	–	–	–	–	–	–	–	–	–	–	–
PC	1	1.80 (1.00–3.24)	0.05	2	1.55 (0.39–6.13)	0.53	–	–	–	1	3.29 (0.63–17.18)	0.16	–	–	–	–	–	–
BL	–	–	–	2	3.61 (1.63–8.02)	0.002	–	–	–	–	–	–	–	–	–	–	–	–
Others	3	1.06 (0.99–1.14)	0.12	4	1.63 (1.07–2.47)	0.02	1	2.02 (0.94–4.36)	0.07	1	1.91 (0.71–5.14)	0.20	–	–	–	–	–	–

**Figure 2:**
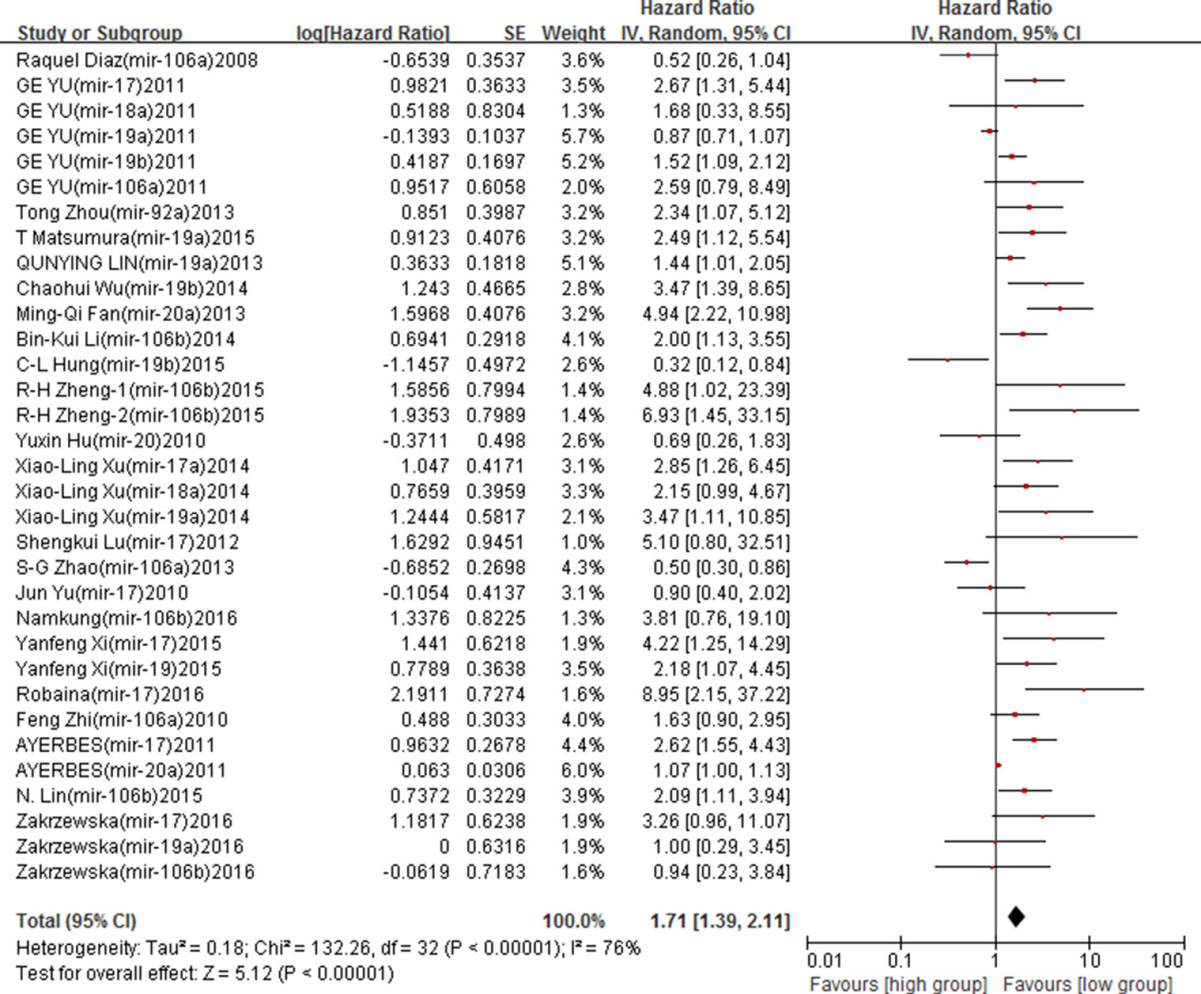
Forest plot of the association between miR-17-92 family and cancer OS – adjusted value

Similar results were found in adjusted analyses. High expression level of miR-17-92 family was an predictor of poor cancer OS (adjusted HRs = 1.71, 95% CIs: 1.39–2.11, *p* < 0.00001). A moderate between-study heterogeneity was found (*I*^2^ = 76%, *p* < 0.00001) (Figure 2). Subgroup analyses by ethnicity found that high expression of miR-17-92 family was associated with poor OS among the Asian (adjusted HRs = 1.91, 95% CIs: 1.45–2.50, *p* < 0.00001). However, no association was found for the Caucasian (adjusted HRs = 1.37, 95% CIs: 0.83–2.26, *p* = 0.22) (Supplementary Figure 1E). Subgroup analyses by sample type, a significant association between high expression of miR-17-92 family and poor OS was found in both tissue (adjusted HRs = 1.68, 95% CIs: 1.35–2.09, *p* < 0.00001) and serum samples (adjusted HRs = 2.20, 95% CIs: 1.08–4.46, *p* = 0.03) (Supplementary Figure 1F). In subgroup analysis by cancer type, high expression of miR-17-92 family was an indicator of poor OS in BC (adjusted HRs = 5.82, 95% CIs:1.92–17.60, *p* = 0.002), ESCC (adjusted HRs = 1.96, 95% CIs:1.01–3.78, *p* = 0.05), BL (adjusted HRs = 3.61, 95% CIs:1.63–8.02, *p* = 0.002) and other cancers (adjusted HRs = 1.63, 95% CIs: 1.07–2.47, *p* = 0.02). No associations was found in CRC, lung cancer, HCC, gliomas and PC (*p* > 0.05) (Supplementary Figure 1G) (Table 3).

**Figure 3:**
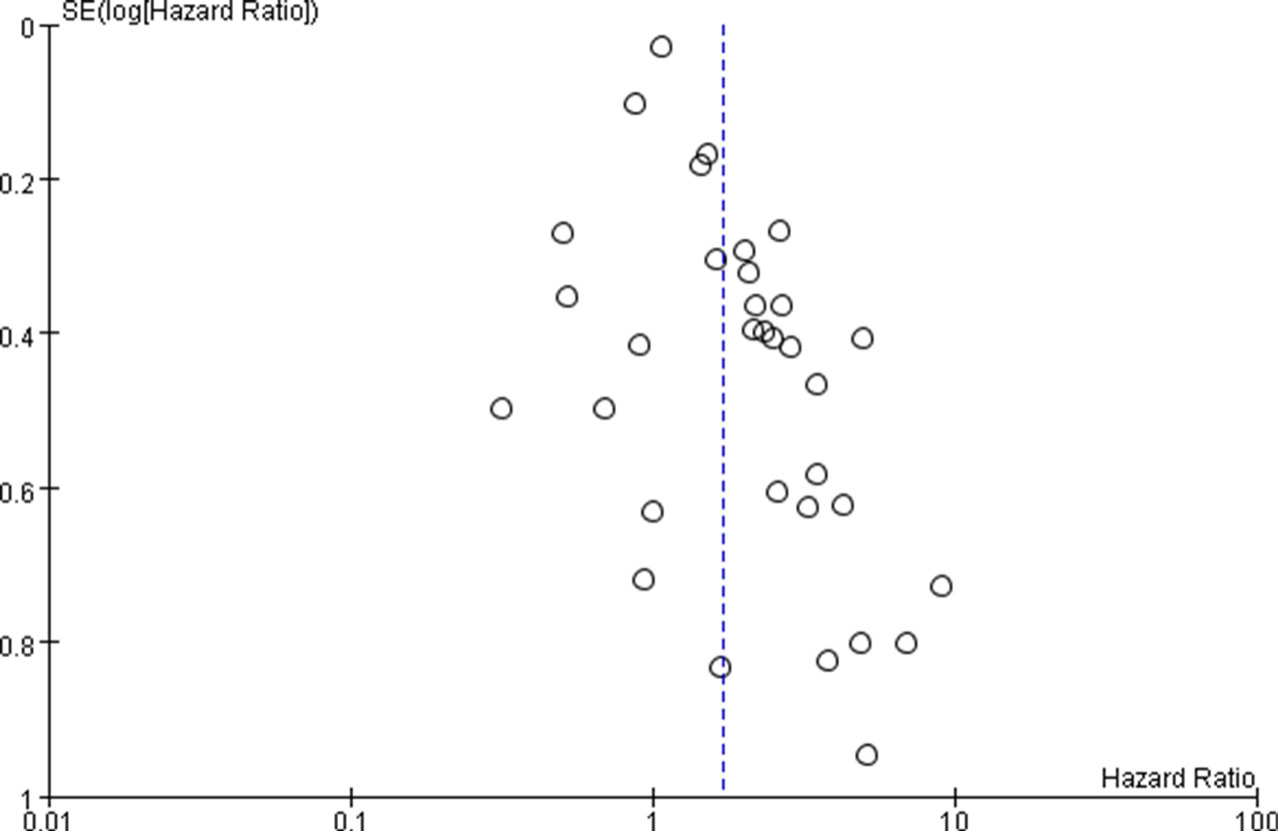
Funnel plot of miR-17-92 family and cancer OS – adjusted value

Funnel plots and Begg's test were used to assess the possibility of publication bias. Funnel plots showed a symmetrical distribution of the points (Figure 3). The *p* value of Beggar's test was 0.403 for OS, suggesting no existing of publication bias in included studies.

### MiR-17-92 family expression and cancer DFS

Ten studies reported the association between miR-17-92 family members and cancer DFS, of which 7 [16, 20, 23–25, 28, 42] studies provided unadjusted DFS values, and 9 [16, 17, 20, 24, 25, 28, 30, 36, 42] reported adjusted values. We pooled unadjusted and adjusted HRs of DFS separately.

In the unadjusted analyses among 7 studies, a significant heterogeneity among studies was observed (*I*^2^ = 87%, *p* < 0.00001), and thus, the random effect model was applied to calculate the pooled HRs and its 95% CIs. Results showed that no association between high expression of miR-17-92 family and cancer DFS (crude HRs = 1.22, 95% CIs: 0.76–1.96, *p* = 0.41) (Supplementary Figure 2A). (Table 3).

**Figure 4:**
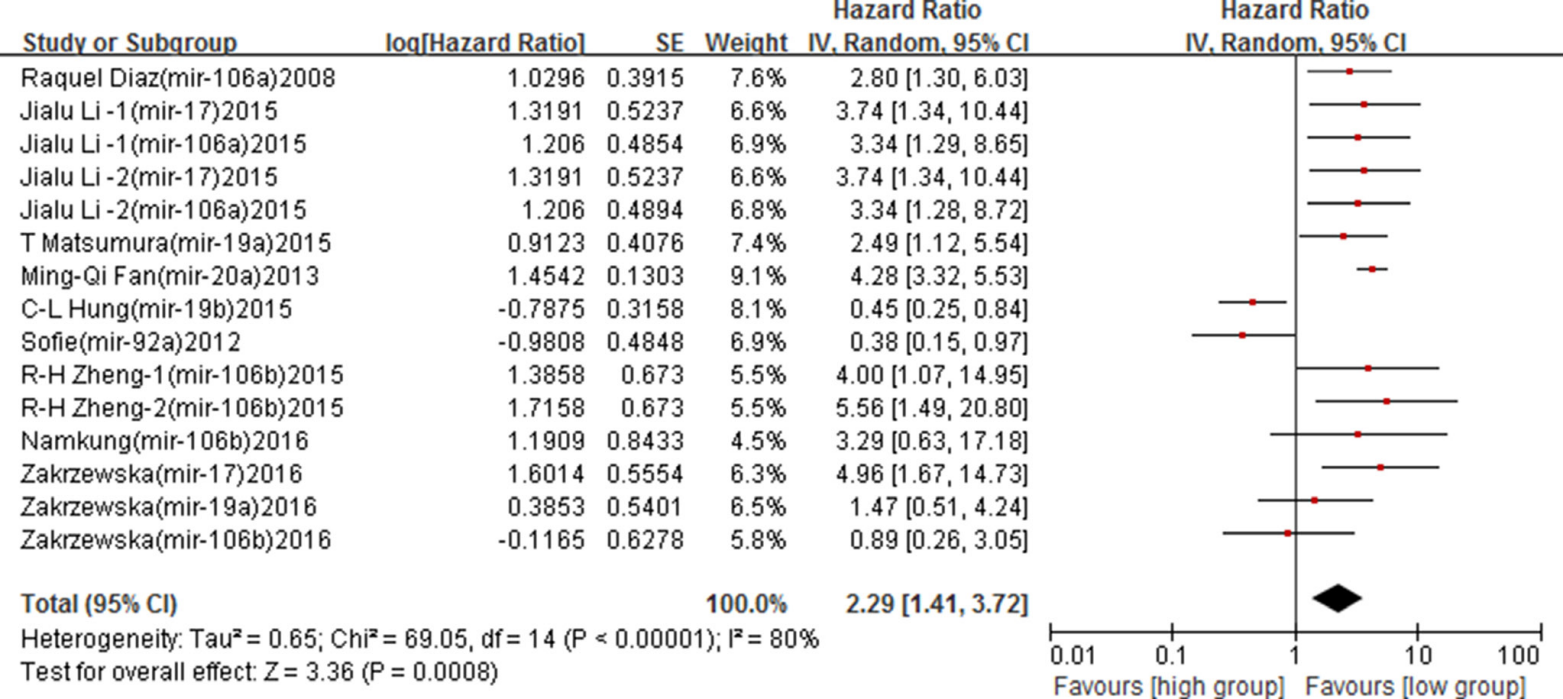
Forest plot of the association between miR-17-92 family and cancer DFS – adjusted value

In the adjusted analyses among 9 studies, contrary results were found. High expression of miR-17-92 family was associated with poor cancer DFS (adjusted HRs = 2.29, 95% CIs: 1.41–3.72, *p* = 0.0008), and a high between-study heterogeneity was found (*I*^2^ = 80%, *p* < 0.00001) (Figure 4). Subgroup analyses based on ethnicity found that high expression of miR-17-92 family was significantly associated with poor DFS among the Asian (adjusted HRs = 2.83, 95% CIs: 1.59–5.04, *p* = 0.0004), while no associations was found in the Caucasian (*p* > 0.05) (Supplementary Figure 2B). In subgroup analysis by sample type, increased expression in serum was significantly associated with poor DFS (adjusted HRs = 3.37, 95% CIs: 2.25–5.02, *p* < 0.00001) (Supplementary Figure 2C) (Table 3).

**Figure 5:**
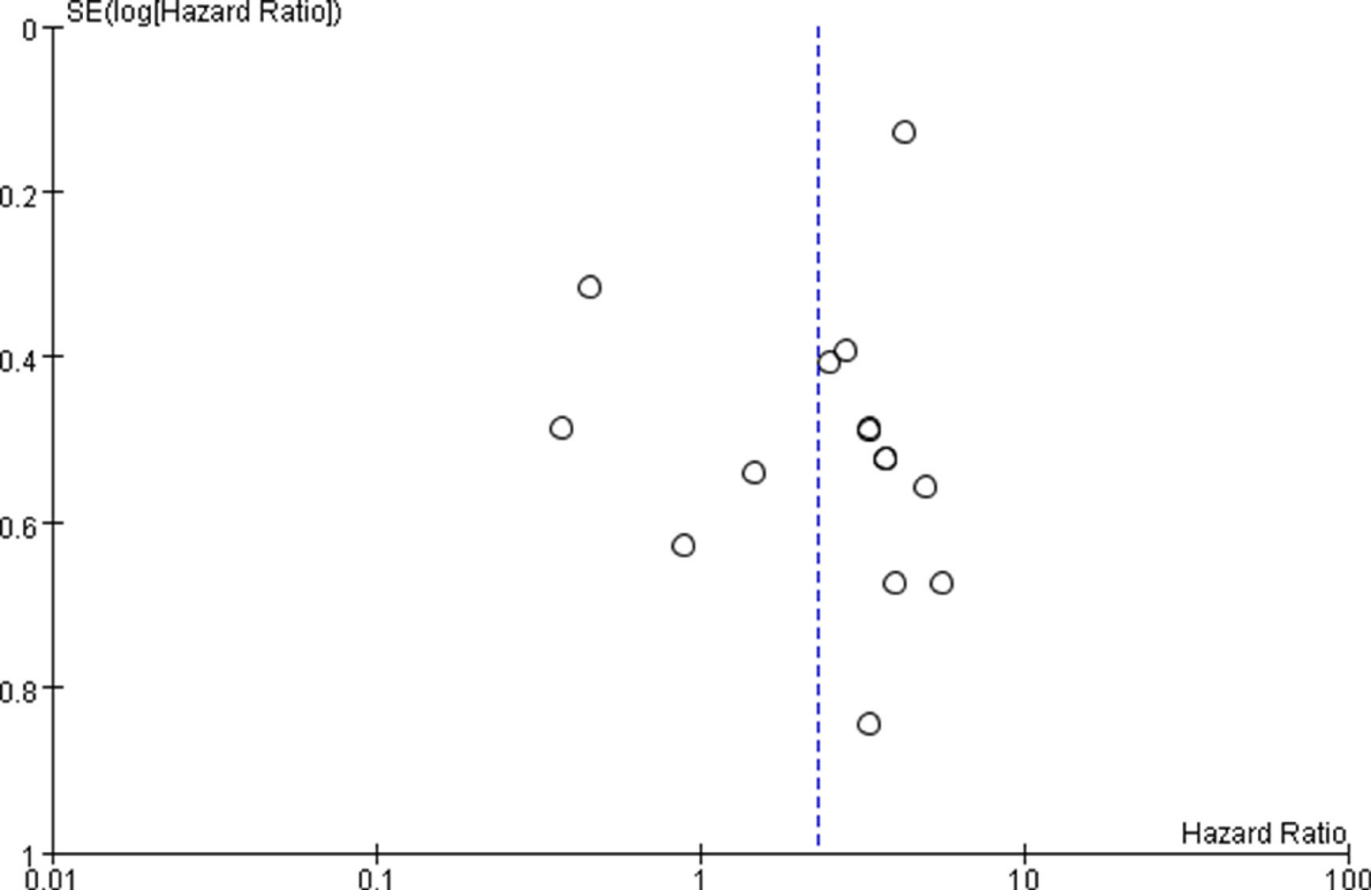
Funnel plot of miR-17-92 family and cancer DFS

No obvious publication bias was found in Begg's test (*Z* = 0.79, *p* = 0.428). The funnel plot also showed a symmetrical distribution, suggesting no publication bias in the overall analysis of included studies (Figure 5).

### MiR-17-92 family expression and cancer PFS

**Figure 6:**
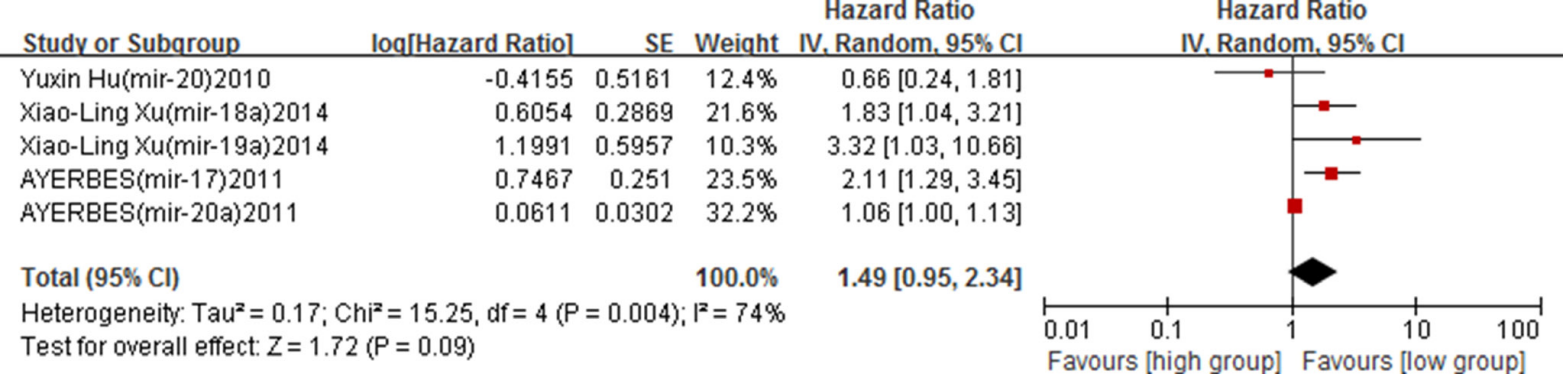
Forest plot of the association between miR-17-92 family and cancer PFS – adjusted value

PFS analyses based on 3 [31, 32, 40] studies showed that high expression of miR-17-92 family was associated with poor cancer PFS (crude HRs = 1.03, 95% CIs: 1.01–1.04, *p* = 0.002). However, no association was found after the adjusted value was calculated (adjusted HRs = 1.49, 95% CIs: 0.95–2.34, *p* = 0.09) (Figure 6). (Table 3).

Only three studies were included to determine the association between the expression of miR-17-92 family and cancer PFS. Thus the publication bias could not well reflect in the funnel plot and Begg's test.

## DISCUSSION

Results of the unadjusted analyses showed that higher expression levels of miR-17-92 family were associated with poor OS and PFS (*p* < 0.05), while no association was found for cancer DFS (*p* > 0.05). In adjusted analyses, high expression of miR-17-92 family was found associated with poor cancer OS and DFS (*p* < 0.05), but no association was found for cancer PFS(*p* > 0.05). Results indicated that increased miR-17-92 family expression plays an important role in cancer OS, and the miR-17-92 family may be a promising biomarker to predict OS in cancer patients. Inconsistent results in PFS and DFS may cause by several reasons. Firstly, pooled cude HRs was calculated from data of univariate analyses of included studies, which did not eliminated effects of confounding factors. The pooled adjusted HRs was computed according to the data of multivariate analyses. Adjusted HRs considered effects of confounders and were more representative than the pooled crude HRs. Secondly, studies included in unadjusted and adjusted analyses were not identical. Some studies reported results of univariate and multivariate analyses simultaneously; while others only presented results of the univariate or multivariate analyses. Thus, we calculated the pooled HRs value separately according to analytical methods used in included studies. Thirdly, confounding factors varied in different studies, which may lead to inconsistency in results. In addition, only 3 studies were included in pooled PFS analysis. Theoretically, the merged data could be affected easily by studies with large sample size. Although some inconsistent results were found in our analysis, the miR-17-92 family still played an important role in cancer prognosis. Inconsistent pooled results implied that the role of miR-17-92 family in cancer prognosis may be complex and controversial.

Results of several previous studies are consistent with our results. A meta-analysis by Jamali et al. [43] found that elevated expressions of miR-18a (HRs = 2.4113, 95% CIs:1.283–4.5289), and miR-19a (HRs = 2.260, *p* = 0.034) was significantly associated with poor survival in patients with human head and neck squamous cell carcinoma (HNSCC). Besides, Zakrzewska et al. [44] found that the miR-17-92 family was important for ependymoma biology. High expression of miR-17 was associated with the increased risk of relapse (HRs = 4.96, 95% CIs:1.67–14.68, *p* = 0.004). Consistent results from different meta-analyses demonstrated that our results are reliable, and that members of miR-17-92 family are important tumour prognostic factors. Detection of miR-17-92 family expression in patients with cancer may be useful for prognosis prediction. However, different results have been published in other meta-analyses. For example, Gu et al. [45] found that increased expression of miR-106b was associated with a favorable prognosis in renal cell carcinoma (HRs = 0.37, 95% CIs:0.15–0.92). The different health outcomes between this study and our meta-analysis may account for the inconsistent results. We explored the prognostic role of miR-17-92 family in several human cancers, while this study merely focued on one member of miR-17-92 family in renal cell carcinoma.

We performed subgroup analyses to assess the effect of ethnicity on OS and DFS. Our results revealed that high expression of miR-17-92 family was significantly associated with poor OS and DFS among the Asian (*p* < 0.05), while no association was found in the Caucasian (*p* > 0.05). This suggests that ethnicity may play an important role in cancer patient prognosis. Similar results have been reported by other studies. Shen et al. [46] noticed that the expression pattern of some miRNAs was sometimes quite different in different pathogenic processes or in different ethnic groups. Also, Hu et al. [47] reported that different ethnic groups had different genetic backgrounds, which may be a potential causes of inconsistency.

Previous studies have reported that abnormal expression of miR-17-92 family was associated with cancer development, such as CRC [48], ESCC [49], and HCC [50]. For example, analysis of the TCGA databases showed that the expression level of miR-17-92 family in HCC tissues were negatively correlated with survival of HCC patients [50]. Results of our sub-analyses by cancer type illustrated that high expression of miR-17-92 family predicted poor OS in BC, ESCC, lymphoma and other cancers. However, no association was found in CRC, lung cancer, HCC, gliomas and PC. Compared with previous studies, parts of our sub-analyses results are consistent with others, while inconsistency also existed. Inconsistent results may be caused by the fact that fewer studies were included in the subgroup analyses, and studies with larger sample size are need to explore the potential function of miR-17-92 family on development and progression of different type of cancers.

**Figure 7:**
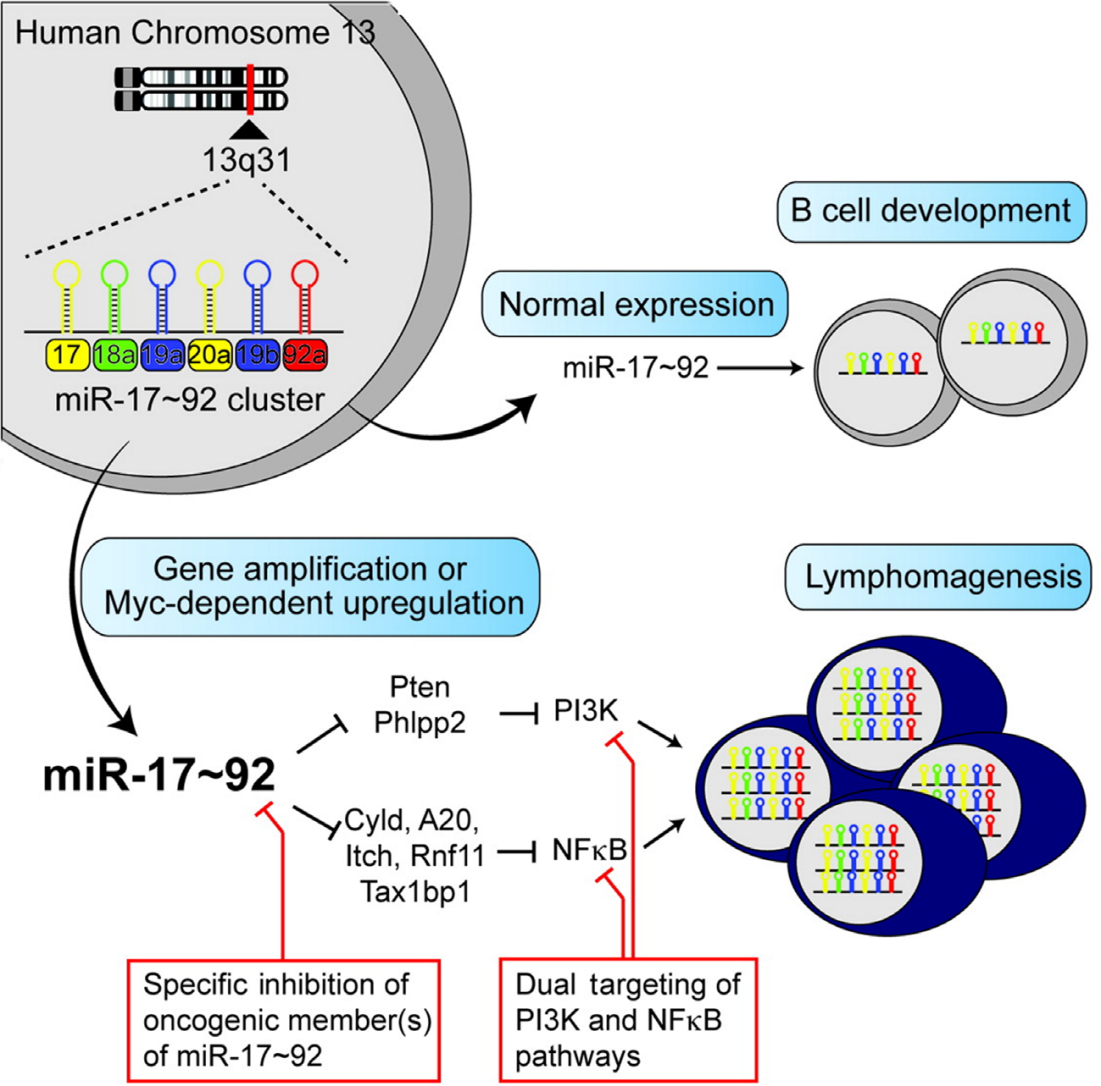
The potential mechanism of MiR-17-92 family in human cancers

The underlying mechanism of miR-17-92 family in cancer prognosis has not been fully understood. Some researchers believed that the function of miR-17-92 family may be concerned with the change in cancer-related proteins and pathways. (Figure 7 [51]). A study conducted by Zhou et al. [52] revealed that miR-17-92 clusters had the function of anti-apoptotic in tumor cells. MiR-17, miR-19, and miR-92 played a role in resistance to apoptosis, since thet could directly inhibit the produced pro-apoptotic proteins through the MAPK/ERK and PI3 K/AKT signaling pathways, which were important in cell survival regulation [52]. Additionally, Kim et al. [53] found that miR-20a could regulate the expression of ZBTB4. Also it was part of a miR-17-92-ZBTB4-Sp transcription factor network that determined the inversely correlated expression of ZBTB4 and Sp1, which were positive and negative prognostic factors, respectively, for survival/relapse-free survival of cancer patients. In addition, Guinot et al. [54] found that the high level of expression of miR-19a/b were targeted and down-regulated the levels of p38a kinase, providing a specific survival signal for Lgr6p cells which mediated by increased Wnt/Δ-catenin activity. Evidence revealed that miR-17-92 family plays a key role in cancer prognosis, which may help with the identification of therapeutic agents for cancer patients.

Compared with above studies, our meta-analyses had several advantages. On one hand, previous studies related to the miR-17-92 family usually analyzed only one or two members; our analyses included more studies, and included all eight miR-17-92 family members instead of a single miRNA. On the other hand, we analyzed the results separately by prognosis outcome (OS/DFS/PFS), which provided a more reliable evidence that miR-17-92 family expression was associated with OS and DFS of human cancers.

However, our study also had several limitations. Firstly, studies included in our analyses were mostly conducted among Asian populations. Sample type and cancer type were incomprehensive as well, which may be important sources of the heterogeneity and inconsistent results found in meta-analyses. Secondly, covariates were different in each included study, which may influence the pooled estimation of the association between miR-17-92 family and cancer prognosis, and our analyses cannot eliminate this impact. Thirdly, factors related to cancer progression such as tumors sizes, stages and metastasis could affect the expression level of miR-17-92 family significantly. However, these factors varied in different studies, which made the sub-analyses exploring effects of these factors impossible in this meta-analyses. Fourthly, though some sub-analyses had been applied, the existing heterogeneity still could not be fully explained. Furthermore, studies providing only survival curves were not included, which might have caused certain degree of bias in our analyses.

In conclusion, despite limitations mentioned above, our results suggest that high miR-17-92 family expression may be an independent risk factor for cancer OS and DFS for the Asian. Also, the type of sample and cancer may be potential sources of heterogeneity. MiR-17-92 family can be potentially used as an non-invasive prognosis biomarkers in clinical therapeutic. Further large-scale prospective studies are needed to explore the potential function of miR-17-92 family in cancer progression.

## MATERIALS AND METHODS

We performed this meta-analysis in accordance with the Preferred Reporting Items for Systematic Reviews and Meta-Analysis guidelines: PRISMA Checklist [55].

### Literature search strategies

Two authors (FFL and HX) independently searched the online PubMed, Cochrane, and Embase databases from their earliest available data to March 31, 2017. The following search terms and combinations were used in keyword and subject heading searches: (“miR-17-92 cluster” OR “miR-17” OR “miR-18a” OR “miR-19a” OR “miR-19b” OR “miR-20a” OR “miR-92a” OR “miR-106a” OR “miR-106b”) and (“neoplasm” OR “neoplasia” OR “cancer” OR “tumor” OR “carcinoma” OR “adenoma”) and (“prognosis” OR “survival” OR “mortality” OR “outcome”). The searches were limited to human studies and articles in English. A manual search was also conducted for the selection of references cited in selected articles.

### Inclusion and exclusion criteria

Literature eligible for inclusion met the following criteria: (1) studies using cohort design reported the association between miR-17-92 family expression and survival outcomes; (2) addressed all the type of cancer; (3) concentrations of miR-17-92 family’ members were detected in serum or tissue; (4) healthy individuals or patients with benign disease were chosen as the control group; (5) reported OS/DFS/PFS with their 95%CIs. We excluded studies if they: (1) were review articles, abstracts, case reports, or letters; (2) were duplicate publications; (3) used cells or adjacent normal tissue as controls; (4) lacked sufficient data; (5) or calculated HRs based on multiple miRNAs.

### Data extraction

Data were extracted from publications independently by two authors (FFL and FZ) according to the inclusion and exclusion criteria above. The characteristics extracted were the name of first author, year of publication, country, sample type, detection method, number of participants, average follow-up time, and HRs of elevated miRNAs for OS, DFS, and PFS, as well as their 95% CIs and *p* values. All the HRs with their 95% CIs and *p* values were collected from the original article. Data were extracted separately when both multivariate and univariate analyses were provided in the same study. Conflicts of data extraction between the two authors were resolved by discussion and consensus with an arbitrator (XYL).

### Quality assessment

The quality of all included studies was systematically assessed by two authors (FFL and FZ) according to the following checklist based on the NOS scale: (1) Selection: including four items [Item 1: Representativeness of the exposed cohort; Item 2: Selection of the non-exposed cohort; Item 3: Ascertainment of exposure; Item 4: Demonstration that outcome of interest was not present at start of study]; (2) Comparability: including one item [Item 5: Comparability of cohorts on the basis of the design or analysis]; and (3) Outcome: including three items [Item 6: Assessment of outcome; Item 7: Was followed up long enough for outcomes to occur; Item 8: Adequacy of follow-up of cohorts]. Total scores of NOS ranged from 0 to 9; studies with NOS scores ≥ 6 were considered as high-quality studies, which studies scores below 6 were regarded low-quality. Any discrepancies were discussed with the third investigator (XYL).

### Statistical analysis

Pooled HRs, 95% CIs, and *p* values were used to estimate the effect across studies for the association between miR-17-92 family and cancer prognosis. Prognosis outcomes mainly contained the OS, DFS, and PFS. Heterogeneity among studies was evaluated using *I*^2^ statistics. The values of *I*^2^ less than 25, 50, and 75% indicated low, moderate, and high degree of heterogeneity, respectively. When significant heterogeneity was observed (*I*^2^ > 50%), pooled HRs estimations were calculated using a random effect model. Otherwise, a fixed effect model was applied [56]. Sensitivity analysis was conducted by removing one study at a time to analyze the influence of individual studies on the summary estimate [57]. Subgroup analyses were conducted based on ethnicity, sample type, and cancer type. The significance of the pooled HRs was determined by the *Z*-test, and a *p* value less than 0.05 was considered statistically significant [58]. Publication bias was assessed with a funnel plot of asymmetry. Begg's test was also performed to assess publication bias [59]. A *p* value less than 0.05 was considered to indicate significant publication bias. All analyses were conducted using RevMan (Version 5.2) software and Stata 12.0.

## SUPPLEMENTARY MATERIALS AND FIGURES


