# Supplementary material for: Prognostic role of miR-17-92 family in human cancers: evaluation of multiple prognostic outcomes
Source: Oncotarget. 2017 Jul 8;8(40):69125–38. doi: 10.18632/oncotarget.19096 (PMC5620325; doi:10.18632/oncotarget.19096)
Supplement: Supplementary file 1 [file oncotarget-08-69125-s001.pdf]

Prognostic role of miR-17-92 family in human cancers: evaluation of multiple prognostic outcomes

Supplementary Materials

A

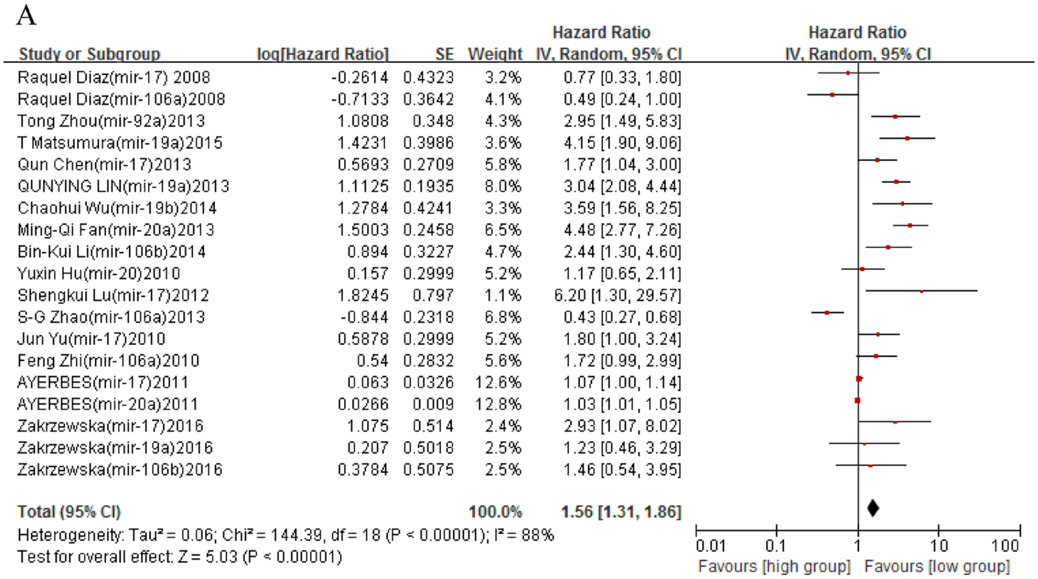

B

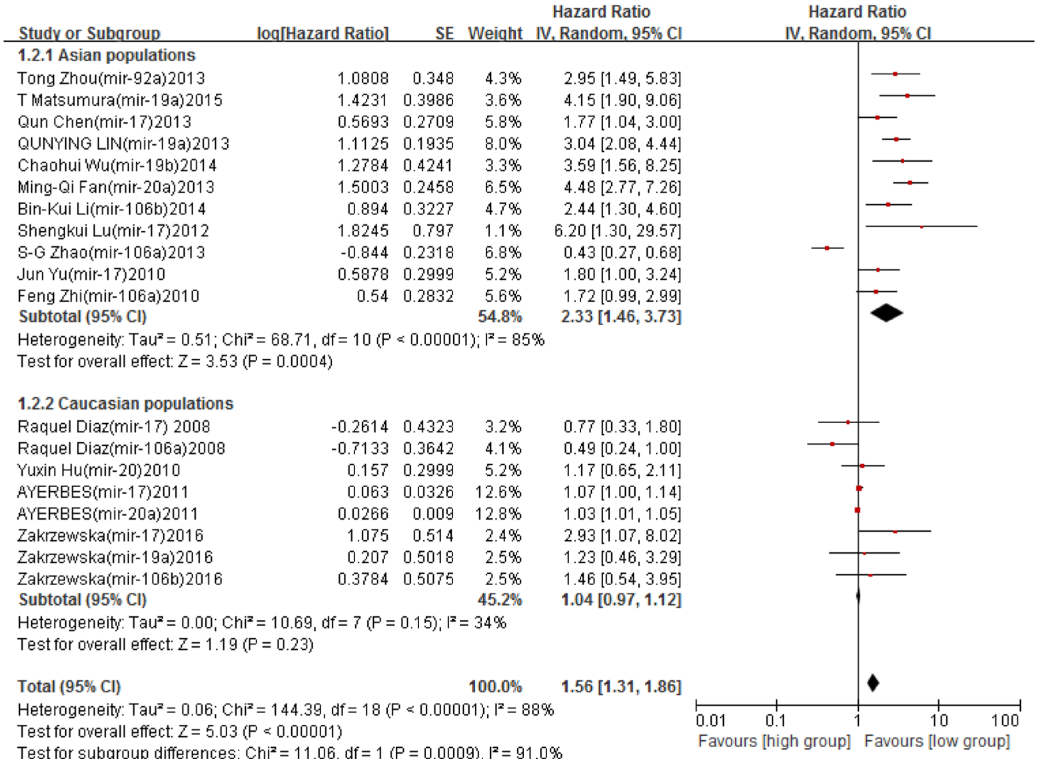

C

Study or Subgroup	log[Hazard Ratio]	SE	Weight	Hazard Ratio IV, Random, 95% CI
**1.3.1 Tissue**				
Raquel Diaz(mir-17) 2008	-0.2614	0.4323	3.2%	0.77 [0.33, 1.80]
Raquel Diaz(mir-106a)2008	-0.7133	0.3642	4.1%	0.49 [0.24, 1.00]
Tong Zhou(mir-92a)2013	1.0808	0.348	4.3%	2.95 [1.49, 5.83]
Chaohui Wu(mir-19b)2014	1.2784	0.4241	3.3%	3.59 [1.56, 8.25]
Ming-Qi Fan(mir-20a)2013	1.5003	0.2458	6.5%	4.48 [2.77, 7.26]
Bin-Kui Li(mir-106b)2014	0.894	0.3227	4.7%	2.44 [1.30, 4.60]
Yuxin Hu(mir-20)2010	0.157	0.2999	5.2%	1.17 [0.65, 2.11]
Shengkui Lu(mir-17)2012	1.8245	0.797	1.1%	6.20 [1.30, 29.57]
S-G Zhao(mir-106a)2013	-0.844	0.2318	6.8%	0.43 [0.27, 0.68]
Jun Yu(mir-17)2010	0.5878	0.2999	5.2%	1.80 [1.00, 3.24]
Feng Zhi(mir-106a)2010	0.54	0.2832	5.6%	1.72 [0.99, 2.99]
AYERBES(mir-17)2011	0.063	0.0326	12.6%	1.07 [1.00, 1.14]
AYERBES(mir-20a)2011	0.0266	0.009	12.8%	1.03 [1.01, 1.05]
Zakrzewska(mir-17)2016	1.075	0.514	2.4%	2.93 [1.07, 8.02]
Zakrzewska(mir-19a)2016	0.207	0.5018	2.5%	1.23 [0.46, 3.29]
Zakrzewska(mir-106b)2016	0.3784	0.5075	2.5%	1.46 [0.54, 3.95]
**Subtotal (95% CI)**			**82.6%**	**1.36 [1.14, 1.61]**
Heterogeneity: Tau² = 0.04; Chi² = 97.32, df = 15 (P < 0.00001); I² = 85%				
Test for overall effect: Z = 3.47 (P = 0.0005)				
**1.3.2 Serum**				
T Matsumura(mir-19a)2015	1.4231	0.3986	3.6%	4.15 [1.90, 9.06]
Qun Chen(mir-17)2013	0.5693	0.2709	5.8%	1.77 [1.04, 3.00]
QUNYING LIN(mir-19a)2013	1.1125	0.1935	8.0%	3.04 [2.08, 4.44]
**Subtotal (95% CI)**			**17.4%**	**2.71 [1.74, 4.20]**
Heterogeneity: Tau² = 0.08; Chi² = 3.99, df = 2 (P = 0.14); I² = 50%				
Test for overall effect: Z = 4.44 (P < 0.00001)				
**Total (95% CI)**			**100.0%**	**1.56 [1.31, 1.86]**
Heterogeneity: Tau² = 0.06; Chi² = 144.39, df = 18 (P < 0.00001); I² = 88%				
Test for overall effect: Z = 5.03 (P < 0.00001)				
Test for subgroup differences: Chi² = 8.24, df = 1 (P = 0.004), I² = 87.9%				

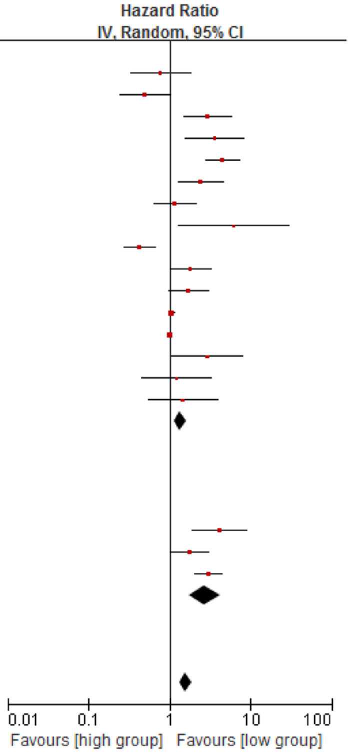

D

Study or Subgroup	log[Hazard Ratio]	SE	Weight	Hazard Ratio IV, Random, 95% CI
**1.4.1 CRC**				
Raquel Diaz(mir-17) 2008	-0.2614	0.4323	3.2%	0.77 [0.33, 1.80]
Raquel Diaz(mir-106a)2008	-0.7133	0.3642	4.1%	0.49 [0.24, 1.00]
Tong Zhou(mir-92a)2013	1.0808	0.348	4.3%	2.95 [1.49, 5.83]
T Matsumura(mir-19a)2015	1.4231	0.3986	3.6%	4.15 [1.90, 9.06]
**Subtotal (95% CI)**			**15.1%**	**1.47 [0.53, 4.08]**
Heterogeneity: Tau² = 0.94; Chi² = 22.10, df = 3 (P < 0.0001); I² = 86%				
Test for overall effect: Z = 0.74 (P = 0.46)				
**1.4.2 Lung cancer**				
Qun Chen(mir-17)2013	0.5693	0.2709	5.8%	1.77 [1.04, 3.00]
QUNYING LIN(mir-19a)2013	1.1125	0.1935	8.0%	3.04 [2.08, 4.44]
Chaohui Wu(mir-19b)2014	1.2784	0.4241	3.3%	3.59 [1.56, 8.25]
**Subtotal (95% CI)**			**17.0%**	**2.61 [1.75, 3.89]**
Heterogeneity: Tau² = 0.05; Chi² = 3.26, df = 2 (P = 0.20); I² = 39%				
Test for overall effect: Z = 4.70 (P < 0.00001)				
**1.4.3 HCC**				
Ming-Qi Fan(mir-20a)2013	1.5003	0.2458	6.5%	4.48 [2.77, 7.26]
Bin-Kui Li(mir-106b)2014	0.894	0.3227	4.7%	2.44 [1.30, 4.60]
**Subtotal (95% CI)**			**11.2%**	**3.43 [1.90, 6.19]**
Heterogeneity: Tau² = 0.10; Chi² = 2.23, df = 1 (P = 0.14); I² = 55%				
Test for overall effect: Z = 4.10 (P < 0.0001)				
**1.4.4 ESCC**				
Yuxin Hu(mir-20)2010	0.157	0.2999	5.2%	1.17 [0.65, 2.11]
**Subtotal (95% CI)**			**5.2%**	**1.17 [0.65, 2.11]**
Heterogeneity: Not applicable				
Test for overall effect: Z = 0.52 (P = 0.60)				
**1.4.5 Glioma**				
Shengkui Lu(mir-17)2012	1.8245	0.797	1.1%	6.20 [1.30, 29.57]
S-G Zhao(mir-106a)2013	-0.844	0.2318	6.8%	0.43 [0.27, 0.68]
**Subtotal (95% CI)**			**8.0%**	**1.46 [0.11, 19.84]**
Heterogeneity: Tau² = 3.22; Chi² = 10.34, df = 1 (P = 0.001); I² = 90%				
Test for overall effect: Z = 0.29 (P = 0.77)				
**1.4.6 PC**				
Jun Yu(mir-17)2010	0.5878	0.2999	5.2%	1.80 [1.00, 3.24]
**Subtotal (95% CI)**			**5.2%**	**1.80 [1.00, 3.24]**
Heterogeneity: Not applicable				
Test for overall effect: Z = 1.96 (P = 0.05)				
**1.4.7 Others**				
Feng Zhi(mir-106a)2010	0.54	0.2832	5.6%	1.72 [0.99, 2.99]
AYERBES(mir-17)2011	0.063	0.0326	12.6%	1.07 [1.00, 1.14]
AYERBES(mir-20a)2011	0.0266	0.009	12.8%	1.03 [1.01, 1.05]
Zakrzewska(mir-17)2016	1.075	0.514	2.4%	2.93 [1.07, 8.02]
Zakrzewska(mir-19a)2016	0.207	0.5018	2.5%	1.23 [0.46, 3.29]
Zakrzewska(mir-106b)2016	0.3784	0.5075	2.5%	1.46 [0.54, 3.95]
**Subtotal (95% CI)**			**38.3%**	**1.06 [0.99, 1.14]**
Heterogeneity: Tau² = 0.00; Chi² = 9.14, df = 5 (P = 0.10); I² = 45%				
Test for overall effect: Z = 1.57 (P = 0.12)				
**Total (95% CI)**			**100.0%**	**1.56 [1.31, 1.86]**
Heterogeneity: Tau² = 0.06; Chi² = 144.39, df = 18 (P < 0.00001); I² = 88%				
Test for overall effect: Z = 5.03 (P < 0.00001)				
Test for subgroup differences: Chi² = 36.16, df = 6 (P < 0.00001), I² = 83.4%				

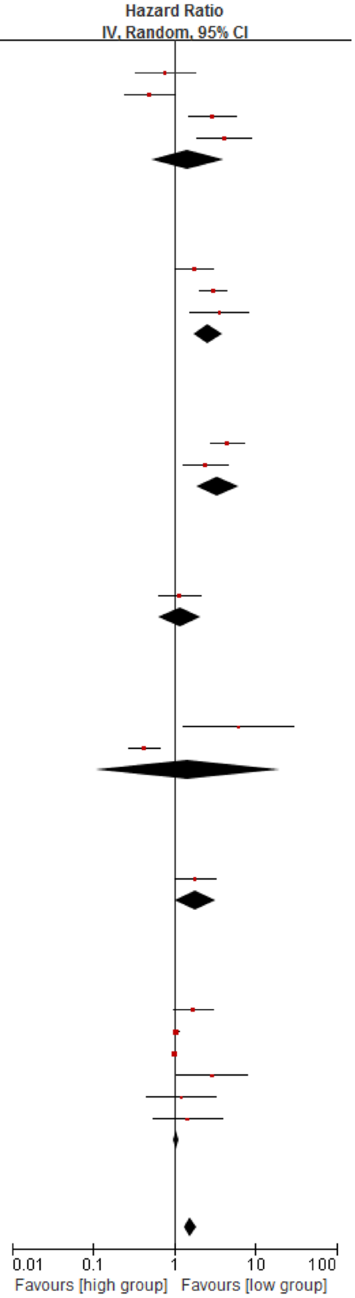

E

Study or Subgroup	log[Hazard Ratio]	SE	Weight	Hazard Ratio IV, Random, 95% CI
**1.6.1 Asian populations**				
GE YU(mir-17)2011	0.9821	0.3633	3.5%	2.67 [1.31, 5.44]
GE YU(mir-18a)2011	0.5188	0.8304	1.3%	1.68 [0.33, 8.55]
GE YU(mir-19a)2011	-0.1393	0.1037	5.7%	0.87 [0.71, 1.07]
GE YU(mir-19b)2011	0.4187	0.1697	5.2%	1.52 [1.09, 2.12]
GE YU(mir-106a)2011	0.9517	0.6058	2.0%	2.59 [0.79, 8.49]
Tong Zhou(mir-92a)2013	0.851	0.3987	3.2%	2.34 [1.07, 5.12]
T Matsumura(mir-19a)2015	0.9123	0.4076	3.2%	2.49 [1.12, 5.54]
QUNYING LIN(mir-19a)2013	0.3633	0.1818	5.1%	1.44 [1.01, 2.05]
Chaohui Wu(mir-19b)2014	1.243	0.4665	2.8%	3.47 [1.39, 8.65]
Ming-Qi Fan(mir-20a)2013	1.5968	0.4076	3.2%	4.94 [2.22, 10.98]
Bin-Kui Li(mir-106b)2014	0.6941	0.2918	4.1%	2.00 [1.13, 3.55]
C-L Hung(mir-19b)2015	-1.1457	0.4972	2.6%	0.32 [0.12, 0.84]
R-H Zheng-1(mir-106b)2015	1.5856	0.7994	1.4%	4.88 [1.02, 23.39]
R-H Zheng-2(mir-106b)2015	1.9353	0.7989	1.4%	6.93 [1.45, 33.15]
Xiao-Ling Xu(mir-17a)2014	0.7659	0.3959	3.3%	2.15 [0.99, 4.67]
Xiao-Ling Xu(mir-18a)2014	1.2444	0.5817	2.1%	3.47 [1.11, 10.85]
Xiao-Ling Xu(mir-19a)2014	1.047	0.4171	3.1%	2.85 [1.26, 6.45]
Shengkui Lu(mir-17)2012	1.6292	0.9451	1.0%	5.10 [0.80, 32.51]
S-G Zhao(mir-106a)2013	-0.6852	0.2698	4.3%	0.50 [0.30, 0.86]
Jun Yu(mir-17)2010	-0.1054	0.4137	3.1%	0.90 [0.40, 2.02]
Namkung(mir-106b)2016	1.3376	0.8225	1.3%	3.81 [0.76, 19.10]
Yanfeng Xi(mir-17)2015	1.441	0.6218	1.9%	4.22 [1.25, 14.29]
Yanfeng Xi(mir-19)2015	0.7789	0.3638	3.5%	2.18 [1.07, 4.45]
Feng Zhi(mir-106a)2010	0.488	0.3033	4.0%	1.63 [0.90, 2.95]
N. Lin(mir-106b)2015	0.7372	0.3229	3.9%	2.09 [1.11, 3.94]
**Subtotal (95% CI)**			**76.4%**	**1.91 [1.45, 2.50]**
Heterogeneity: Tau² = 0.28; Chi² = 91.06, df = 24 (P < 0.00001); I² = 74%				
Test for overall effect: Z = 4.68 (P < 0.00001)				
**1.6.2 Caucasian populations**				
Raquel Diaz(mir-106a)2008	-0.6539	0.3537	3.6%	0.52 [0.26, 1.04]
Yuxin Hu(mir-20)2010	-0.3711	0.498	2.6%	0.69 [0.26, 1.83]
Robaina(mir-17)2016	2.1911	0.7274	1.6%	8.95 [2.15, 37.22]
AYERBES(mir-17)2011	0.9632	0.2678	4.4%	2.62 [1.55, 4.43]
AYERBES(mir-20a)2011	0.063	0.0306	6.0%	1.07 [1.00, 1.13]
Zakrzewska(mir-17)2016	1.1817	0.6238	1.9%	3.26 [0.96, 11.07]
Zakrzewska(mir-19a)2016	0	0.6316	1.9%	1.00 [0.29, 3.45]
Zakrzewska(mir-106b)2016	-0.0619	0.7183	1.6%	0.94 [0.23, 3.84]
**Subtotal (95% CI)**			**23.6%**	**1.37 [0.83, 2.26]**
Heterogeneity: Tau² = 0.31; Chi² = 27.86, df = 7 (P = 0.0002); I² = 75%				
Test for overall effect: Z = 1.23 (P = 0.22)				
**Total (95% CI)**			**100.0%**	**1.71 [1.39, 2.11]**
Heterogeneity: Tau² = 0.18; Chi² = 132.26, df = 32 (P < 0.00001); I² = 76%				
Test for overall effect: Z = 5.12 (P < 0.00001)				
Test for subgroup differences: Chi² = 1.30, df = 1 (P = 0.25), I² = 23.0%				

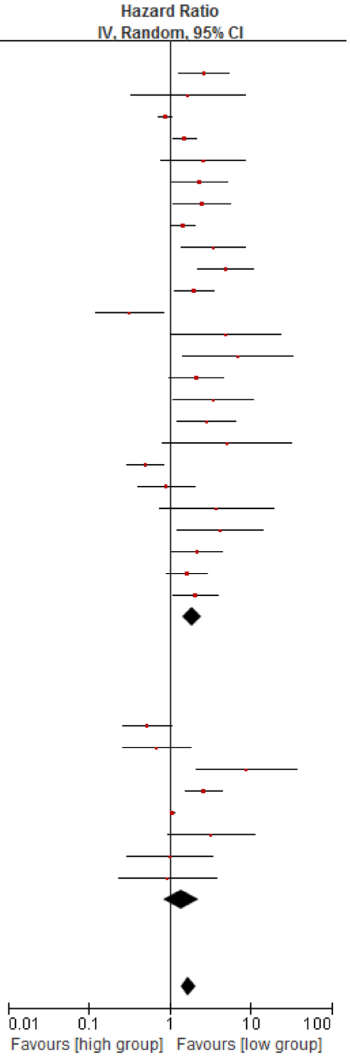

F

Study or Subgroup	log[Hazard Ratio]	SE	Weight	Hazard Ratio IV, Random, 95% CI
**1.7.1 Tissue**				
Raquel Diaz(mir-106a)2008	-0.6539	0.3537	3.6%	0.52 [0.26, 1.04]
GE YU(mir-17)2011	0.9821	0.3633	3.5%	2.67 [1.31, 5.44]
GE YU(mir-18a)2011	0.5188	0.8304	1.3%	1.68 [0.33, 8.55]
GE YU(mir-19a)2011	-0.1393	0.1037	5.7%	0.87 [0.71, 1.07]
GE YU(mir-19b)2011	0.4187	0.1697	5.2%	1.52 [1.09, 2.12]
GE YU(mir-106a)2011	0.9517	0.6058	2.0%	2.59 [0.79, 8.49]
Tong Zhou(mir-92a)2013	0.851	0.3987	3.2%	2.34 [1.07, 5.12]
Chaohui Wu(mir-19b)2014	1.243	0.4665	2.8%	3.47 [1.39, 8.65]
Ming-Qi Fan(mir-20a)2013	1.5968	0.4076	3.2%	4.94 [2.22, 10.98]
Bin-Kui Li(mir-106b)2014	0.6941	0.2918	4.1%	2.00 [1.13, 3.55]
C-L Hung(mir-19b)2015	-1.1457	0.4972	2.6%	0.32 [0.12, 0.84]
R-H Zheng-1(mir-106b)2015	1.5856	0.7994	1.4%	4.88 [1.02, 23.39]
Yuxin Hu(mir-20)2010	-0.3711	0.498	2.6%	0.69 [0.26, 1.83]
Xiao-Ling Xu(mir-17a)2014	1.047	0.4171	3.1%	2.85 [1.26, 6.45]
Xiao-Ling Xu(mir-18a)2014	0.7659	0.3959	3.3%	2.15 [0.99, 4.67]
Xiao-Ling Xu(mir-19a)2014	1.2444	0.5817	2.1%	3.47 [1.11, 10.85]
Shengkui Lu(mir-17)2012	1.6292	0.9451	1.0%	5.10 [0.80, 32.51]
S-G Zhao(mir-106a)2013	-0.6852	0.2698	4.3%	0.50 [0.30, 0.86]
Jun Yu(mir-17)2010	-0.1054	0.4137	3.1%	0.90 [0.40, 2.02]
Namkung(mir-106b)2016	1.3376	0.8225	1.3%	3.81 [0.76, 19.10]
Yanfeng Xi(mir-17)2015	1.441	0.6218	1.9%	4.22 [1.25, 14.29]
Yanfeng Xi(mir-19)2015	0.7789	0.3638	3.5%	2.18 [1.07, 4.45]
Robaina(mir-17)2016	2.1911	0.7274	1.6%	8.95 [2.15, 37.22]
Feng Zhi(mir-106a)2010	0.488	0.3033	4.0%	1.63 [0.90, 2.95]
AYERBES(mir-17)2011	0.9632	0.2678	4.4%	2.62 [1.55, 4.43]
AYERBES(mir-20a)2011	0.063	0.0306	6.0%	1.07 [1.00, 1.13]
N. Lin(mir-106b)2015	0.7372	0.3229	3.9%	2.09 [1.11, 3.94]
Zakrzewska(mir-17)2016	1.1817	0.6238	1.9%	3.26 [0.96, 11.07]
Zakrzewska(mir-19a)2016	0	0.6316	1.9%	1.00 [0.29, 3.45]
Zakrzewska(mir-106b)2016	-0.0619	0.7183	1.6%	0.94 [0.23, 3.84]
**Subtotal (95% CI)**			**90.3%**	**1.68 [1.35, 2.09]**
Heterogeneity: Tau² = 0.19; Chi² = 121.45, df = 29 (P < 0.00001); I² = 76%				
Test for overall effect: Z = 4.62 (P < 0.00001)				
**1.7.2 Serum**				
T Matsumura(mir-19a)2015	0.9123	0.4076	3.2%	2.49 [1.12, 5.54]
QUNYING LIN(mir-19a)2013	0.3633	0.1818	5.1%	1.44 [1.01, 2.05]
R-H Zheng-2(mir-106b)2015	1.9353	0.7989	1.4%	6.93 [1.45, 33.15]
**Subtotal (95% CI)**			**9.7%**	**2.20 [1.08, 4.46]**
Heterogeneity: Tau² = 0.22; Chi² = 4.81, df = 2 (P = 0.09); I² = 58%				
Test for overall effect: Z = 2.18 (P = 0.03)				
**Total (95% CI)**			**100.0%**	**1.71 [1.39, 2.11]**
Heterogeneity: Tau² = 0.18; Chi² = 132.26, df = 32 (P < 0.00001); I² = 76%				
Test for overall effect: Z = 5.12 (P < 0.00001)				
Test for subgroup differences: Chi² = 0.51, df = 1 (P = 0.48), I² = 0%				

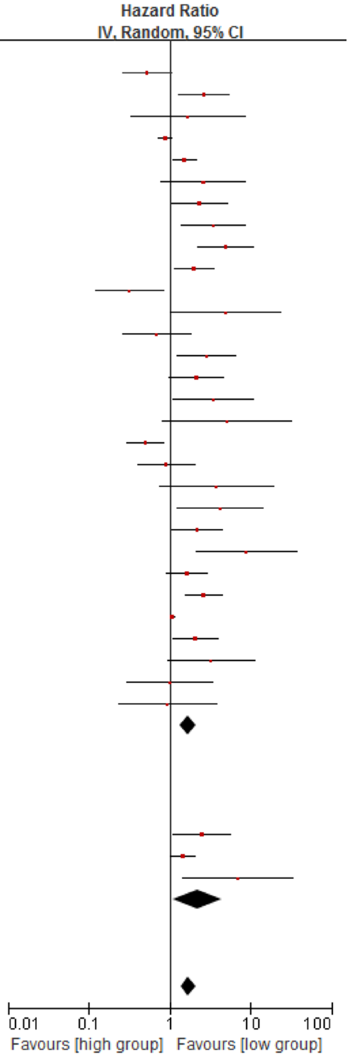

G

Study or Subgroup	log[Hazard Ratio]	SE	Weight	Hazard Ratio IV, Random, 95% CI
**1.8.1 CRC**				
Raquel Diaz(mir-106a)2008	-0.6539	0.3537	3.6%	0.52 [0.26, 1.04]
GE YU(mir-17)2011	0.9821	0.3633	3.5%	2.67 [1.31, 5.44]
GE YU(mir-18a)2011	0.5188	0.8304	1.3%	1.68 [0.33, 8.55]
GE YU(mir-19a)2011	-0.1393	0.1037	5.7%	0.87 [0.71, 1.07]
GE YU(mir-19b)2011	0.4187	0.1697	5.2%	1.52 [1.09, 2.12]
GE YU(mir-106a)2011	0.9517	0.6058	2.0%	2.59 [0.79, 8.49]
Tong Zhou(mir-92a)2013	0.851	0.3987	3.2%	2.34 [1.07, 5.12]
T Matsumura(mir-19a)2015	0.9123	0.4076	3.2%	2.49 [1.12, 5.54]
**Subtotal (95% CI)**			**27.8%**	**1.49 [0.98, 2.27]**
Heterogeneity: Tau² = 0.23; Chi² = 29.02, df = 7 (P = 0.0001); I² = 76%				
Test for overall effect: Z = 1.85 (P = 0.06)				
**1.8.2 Lung cancer**				
QUNYING LIN(mir-19a)2013	0.3633	0.1818	5.1%	1.44 [1.01, 2.05]
Chaohui Wu(mir-19b)2014	1.243	0.4665	2.8%	3.47 [1.39, 8.65]
**Subtotal (95% CI)**			**7.9%**	**2.01 [0.87, 4.64]**
Heterogeneity: Tau² = 0.26; Chi² = 3.09, df = 1 (P = 0.08); I² = 68%				
Test for overall effect: Z = 1.63 (P = 0.10)				
**1.8.3 HCC**				
Ming-Qi Fan(mir-20a)2013	1.5968	0.4076	3.2%	4.94 [2.22, 10.98]
Bin-Kui Li(mir-106b)2014	0.6941	0.2918	4.1%	2.00 [1.13, 3.55]
C-L Hung(mir-19b)2015	-1.1457	0.4972	2.6%	0.32 [0.12, 0.84]
**Subtotal (95% CI)**			**9.9%**	**1.52 [0.39, 5.83]**
Heterogeneity: Tau² = 1.26; Chi² = 18.41, df = 2 (P = 0.0001); I² = 89%				
Test for overall effect: Z = 0.60 (P = 0.55)				
**1.8.4 BC**				
R-H Zheng-1(mir-106b)2015	1.5856	0.7994	1.4%	4.88 [1.02, 23.39]
R-H Zheng-2(mir-106b)2015	1.9353	0.7989	1.4%	6.93 [1.45, 33.15]
**Subtotal (95% CI)**			**2.7%**	**5.82 [1.92, 17.60]**
Heterogeneity: Tau² = 0.00; Chi² = 0.10, df = 1 (P = 0.76); I² = 0%				
Test for overall effect: Z = 3.12 (P = 0.002)				
**1.8.5 ESCC**				
Yuxin Hu(mir-20)2010	-0.3711	0.498	2.6%	0.69 [0.26, 1.83]
Xiao-Ling Xu(mir-17a)2014	1.047	0.4171	3.1%	2.85 [1.26, 6.45]
Xiao-Ling Xu(mir-18a)2014	0.7659	0.3959	3.3%	2.15 [0.99, 4.67]
Xiao-Ling Xu(mir-19a)2014	1.2444	0.5817	2.1%	3.47 [1.11, 10.85]
**Subtotal (95% CI)**			**11.1%**	**1.96 [1.01, 3.78]**
Heterogeneity: Tau² = 0.23; Chi² = 6.22, df = 3 (P = 0.10); I² = 52%				
Test for overall effect: Z = 2.00 (P = 0.05)				
**1.8.6 Glioma**				
Shengkui Lu(mir-17)2012	1.6292	0.9451	1.0%	5.10 [0.80, 32.51]
S-G Zhao(mir-106a)2013	-0.6852	0.2698	4.3%	0.50 [0.30, 0.86]
**Subtotal (95% CI)**			**5.4%**	**1.34 [0.14, 12.63]**
Heterogeneity: Tau² = 2.20; Chi² = 5.54, df = 1 (P = 0.02); I² = 82%				
Test for overall effect: Z = 0.26 (P = 0.80)				
**1.8.7 PC**				
Jun Yu(mir-17)2010	-0.1054	0.4137	3.1%	0.90 [0.40, 2.02]
Namkung(mir-106b)2016	1.3376	0.8225	1.3%	3.81 [0.76, 19.10]
**Subtotal (95% CI)**			**4.4%**	**1.55 [0.39, 6.13]**
Heterogeneity: Tau² = 0.62; Chi² = 2.46, df = 1 (P = 0.12); I² = 59%				
Test for overall effect: Z = 0.63 (P = 0.53)				
**1.8.8 BL**				
Yanfeng Xi(mir-17)2015	1.441	0.6218	1.9%	4.22 [1.25, 14.29]
Yanfeng Xi(mir-19)2015	0.7789	0.3638	3.5%	2.18 [1.07, 4.45]
Robaina(mir-17)2016	2.1911	0.7274	1.6%	8.95 [2.15, 37.22]
**Subtotal (95% CI)**			**7.0%**	**3.61 [1.63, 8.02]**
Heterogeneity: Tau² = 0.20; Chi² = 3.31, df = 2 (P = 0.19); I² = 40%				
Test for overall effect: Z = 3.16 (P = 0.002)				
**1.8.9 Others**				
Feng Zhi(mir-106a)2010	0.488	0.3033	4.0%	1.63 [0.90, 2.95]
AYERBES(mir-17)2011	0.9632	0.2678	4.4%	2.62 [1.55, 4.43]
AYERBES(mir-20a)2011	0.063	0.0306	6.0%	1.07 [1.00, 1.13]
N. Lin(mir-106b)2015	0.7372	0.3229	3.9%	2.09 [1.11, 3.94]
Zakrzewska(mir-17)2016	1.1817	0.6238	1.9%	3.26 [0.96, 11.07]
Zakrzewska(mir-19a)2016	0	0.6316	1.9%	1.00 [0.29, 3.45]
Zakrzewska(mir-106b)2016	-0.0619	0.7183	1.6%	0.94 [0.23, 3.84]
**Subtotal (95% CI)**			**23.7%**	**1.63 [1.07, 2.47]**
Heterogeneity: Tau² = 0.18; Chi² = 20.26, df = 6 (P = 0.002); I² = 70%				
Test for overall effect: Z = 2.28 (P = 0.02)				
**Total (95% CI)**			**100.0%**	**1.71 [1.39, 2.11]**
Heterogeneity: Tau² = 0.18; Chi² = 132.26, df = 32 (P < 0.00001); I² = 76%				
Test for overall effect: Z = 5.12 (P < 0.00001)				
Test for subgroup differences: Chi² = 8.47, df = 8 (P = 0.39), I² = 5.6%				

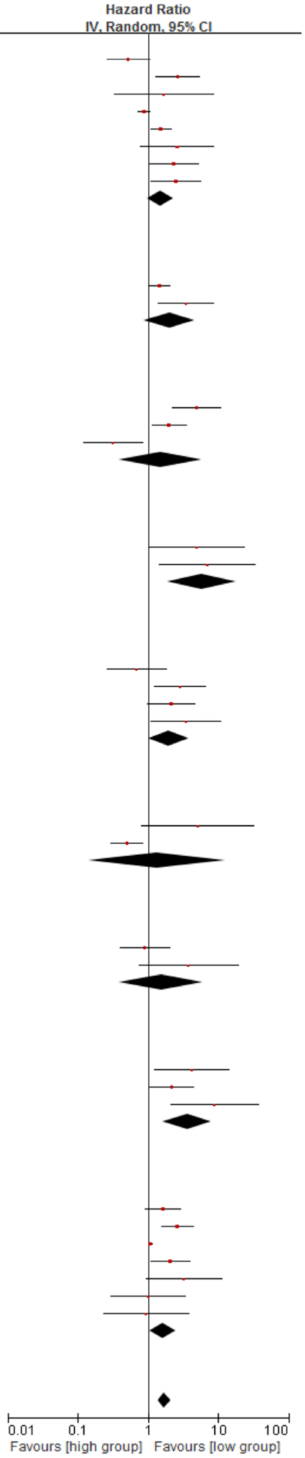

Supplementary Figure 1: (**A**) Forest plot of the association between miR-17-92 family and cancer OS-unadjusted value. (**B**) Forest plot of the association between miR-17-92 family and cancer OS-unadjusted value subgroup analysis (Asian vs Caucasian). (**C**) Forest plot of the association between miR-17-92 family and cancer OS-unadjusted value subgroup analysis (Tissue sample vs Serum sample). (**D**) Forest plot of the association between miR-17-92 family and cancer OS-unadjusted value subgroup analysis (cancer type). (**E**) Forest plot of the association between miR-17-92 family and cancer OS-adjusted value subgroup analysis (Asian vs Caucasian). (**F**) Forest plot of the association between miR-17-92 family and cancer OS-adjusted value subgroup analysis (Tissue sample vs Serum sample). (**G**) Forest plot of the association between miR-17-92 family and cancer OS-adjusted value subgroup analysis (cancer type).

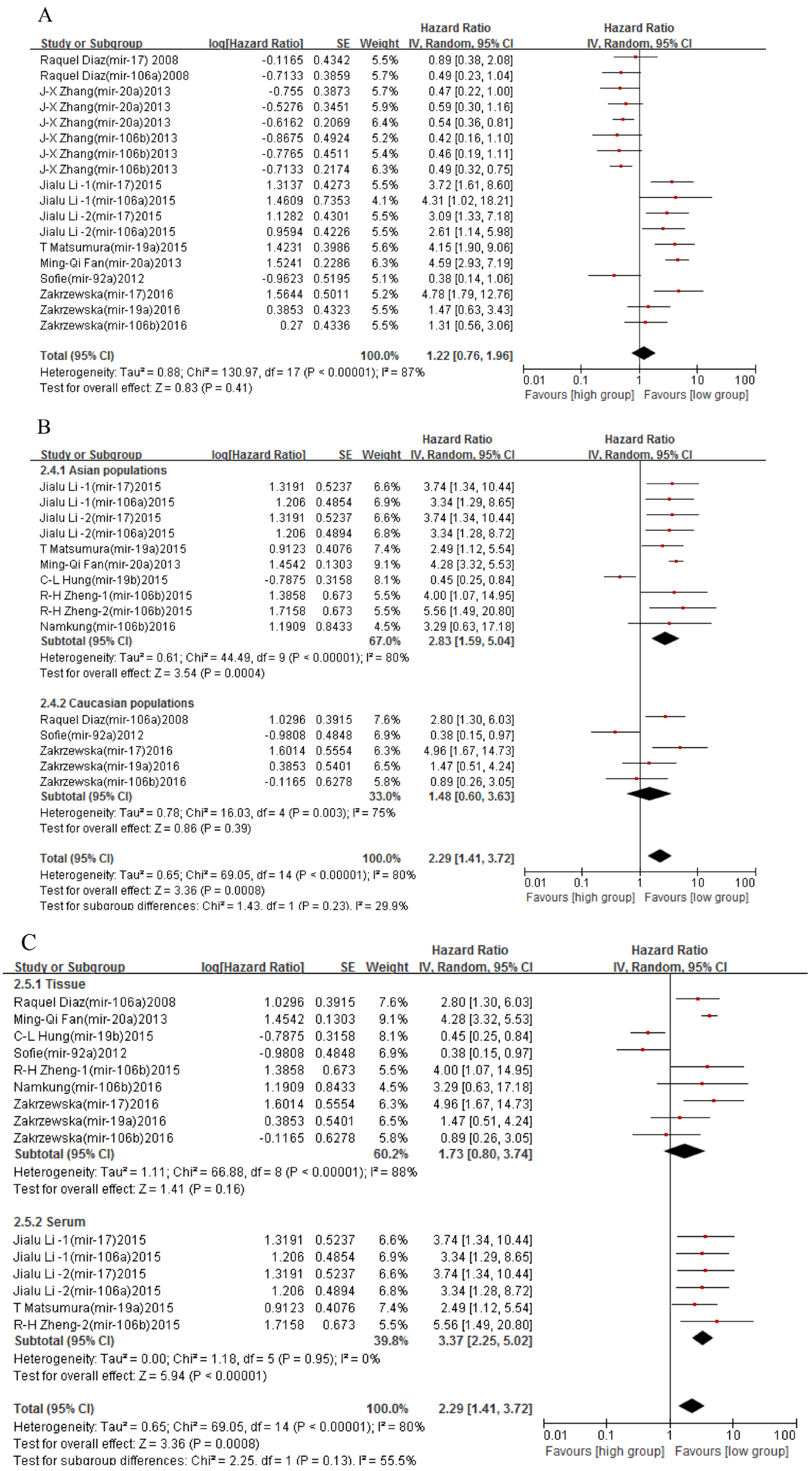
A
Study or Subgroup log[Hazard Ratio] SE Weight Hazard Ratio IV, Random, 95% CI Hazard Ratio IV, Random, 95% CI
Raquel Diaz(mir-17) 2008 -0.1165 0.4342 5.5% 0.89 [0.38, 2.08]
Raquel Diaz(mir-106a)2008 -0.7133 0.3859 5.7% 0.49 [0.23, 1.04]
J-X Zhang(mir-20a)2013 -0.755 0.3873 5.7% 0.47 [0.22, 1.00]
J-X Zhang(mir-20a)2013 -0.5276 0.3451 5.9% 0.59 [0.30, 1.16]
J-X Zhang(mir-20a)2013 -0.6162 0.2069 6.4% 0.54 [0.36, 0.81]
J-X Zhang(mir-106b)2013 -0.8675 0.4924 5.2% 0.42 [0.16, 1.10]
J-X Zhang(mir-106b)2013 -0.7765 0.4511 5.4% 0.46 [0.19, 1.11]
J-X Zhang(mir-106b)2013 -0.7133 0.2174 6.3% 0.49 [0.32, 0.75]
Jialu Li -1(mir-17)2015 1.3137 0.4273 5.5% 3.72 [1.61, 8.60]
Jialu Li -1(mir-106a)2015 1.4609 0.7353 4.1% 4.31 [1.02, 18.21]
Jialu Li -2(mir-17)2015 1.1282 0.4301 5.5% 3.09 [1.33, 7.18]
Jialu Li -2(mir-106a)2015 0.9594 0.4226 5.5% 2.61 [1.14, 5.98]
T Matsumura(mir-19a)2015 1.4231 0.3986 5.6% 4.15 [1.90, 9.06]
Ming-Qi Fan(mir-20a)2013 1.5241 0.2286 6.3% 4.59 [2.93, 7.19]
Sofie(mir-92a)2012 -0.9623 0.5195 5.1% 0.38 [0.14, 1.06]
Zakrzewska(mir-17)2016 1.5644 0.5011 5.2% 4.78 [1.79, 12.76]
Zakrzewska(mir-19a)2016 0.3853 0.4323 5.5% 1.47 [0.63, 3.43]
Zakrzewska(mir-106b)2016 0.27 0.4336 5.5% 1.31 [0.56, 3.06]
Total (95% CI) 100.0% 1.22 [0.76, 1.96]
Heterogeneity: Tau² = 0.88; Chi² = 130.97, df = 17 (P < 0.00001); I² = 87%
Test for overall effect: Z = 0.83 (P = 0.41)
0.01 0.1 1 10 100
Favours [high group] Favours [low group]
B
Study or Subgroup log[Hazard Ratio] SE Weight Hazard Ratio IV, Random, 95% CI Hazard Ratio IV, Random, 95% CI
2.4.1 Asian populations
Jialu Li -1(mir-17)2015 1.3191 0.5237 6.6% 3.74 [1.34, 10.44]
Jialu Li -1(mir-106a)2015 1.206 0.4854 6.9% 3.34 [1.29, 8.65]
Jialu Li -2(mir-17)2015 1.3191 0.5237 6.6% 3.74 [1.34, 10.44]
Jialu Li -2(mir-106a)2015 1.206 0.4894 6.8% 3.34 [1.28, 8.72]
T Matsumura(mir-19a)2015 0.9123 0.4076 7.4% 2.49 [1.12, 5.54]
Ming-Qi Fan(mir-20a)2013 1.4542 0.1303 9.1% 4.28 [3.32, 5.53]
C-L Hung(mir-19b)2015 -0.7875 0.3158 8.1% 0.45 [0.25, 0.84]
R-H Zheng-1(mir-106b)2015 1.3858 0.673 5.5% 4.00 [1.07, 14.95]
R-H Zheng-2(mir-106b)2015 1.7158 0.673 5.5% 5.56 [1.49, 20.80]
Namkung(mir-106b)2016 1.1909 0.8433 4.5% 3.29 [0.63, 17.18]
Subtotal (95% CI) 67.0% 2.83 [1.59, 5.04]
Heterogeneity: Tau² = 0.61; Chi² = 44.49, df = 9 (P < 0.00001); I² = 80%
Test for overall effect: Z = 3.54 (P = 0.0004)
2.4.2 Caucasian populations
Raquel Diaz(mir-106a)2008 1.0296 0.3915 7.6% 2.80 [1.30, 6.03]
Sofie(mir-92a)2012 -0.9808 0.4848 6.9% 0.38 [0.15, 0.97]
Zakrzewska(mir-17)2016 1.6014 0.5554 6.3% 4.96 [1.67, 14.73]
Zakrzewska(mir-19a)2016 0.3853 0.5401 6.5% 1.47 [0.51, 4.24]
Zakrzewska(mir-106b)2016 -0.1165 0.6278 5.8% 0.89 [0.26, 3.05]
Subtotal (95% CI) 33.0% 1.48 [0.60, 3.63]
Heterogeneity: Tau² = 0.78; Chi² = 16.03, df = 4 (P = 0.003); I² = 75%
Test for overall effect: Z = 0.86 (P = 0.39)
Total (95% CI) 100.0% 2.29 [1.41, 3.72]
Heterogeneity: Tau² = 0.65; Chi² = 69.05, df = 14 (P < 0.00001); I² = 80%
Test for overall effect: Z = 3.36 (P = 0.0008)
Test for subgroup differences: Chi² = 1.43, df = 1 (P = 0.23), I² = 29.9%
0.01 0.1 1 10 100
Favours [high group] Favours [low group]
C
Study or Subgroup log[Hazard Ratio] SE Weight Hazard Ratio IV, Random, 95% CI Hazard Ratio IV, Random, 95% CI
2.5.1 Tissue
Raquel Diaz(mir-106a)2008 1.0296 0.3915 7.6% 2.80 [1.30, 6.03]
Ming-Qi Fan(mir-20a)2013 1.4542 0.1303 9.1% 4.28 [3.32, 5.53]
C-L Hung(mir-19b)2015 -0.7875 0.3158 8.1% 0.45 [0.25, 0.84]
Sofie(mir-92a)2012 -0.9808 0.4848 6.9% 0.38 [0.15, 0.97]
R-H Zheng-1(mir-106b)2015 1.3858 0.673 5.5% 4.00 [1.07, 14.95]
Namkung(mir-106b)2016 1.1909 0.8433 4.5% 3.29 [0.63, 17.18]
Zakrzewska(mir-17)2016 1.6014 0.5554 6.3% 4.96 [1.67, 14.73]
Zakrzewska(mir-19a)2016 0.3853 0.5401 6.5% 1.47 [0.51, 4.24]
Zakrzewska(mir-106b)2016 -0.1165 0.6278 5.8% 0.89 [0.26, 3.05]
Subtotal (95% CI) 60.2% 1.73 [0.80, 3.74]
Heterogeneity: Tau² = 1.11; Chi² = 66.88, df = 8 (P < 0.00001); I² = 88%
Test for overall effect: Z = 1.41 (P = 0.16)
2.5.2 Serum
Jialu Li -1(mir-17)2015 1.3191 0.5237 6.6% 3.74 [1.34, 10.44]
Jialu Li -1(mir-106a)2015 1.206 0.4854 6.9% 3.34 [1.29, 8.65]
Jialu Li -2(mir-17)2015 1.3191 0.5237 6.6% 3.74 [1.34, 10.44]
Jialu Li -2(mir-106a)2015 1.206 0.4894 6.8% 3.34 [1.28, 8.72]
T Matsumura(mir-19a)2015 0.9123 0.4076 7.4% 2.49 [1.12, 5.54]
R-H Zheng-2(mir-106b)2015 1.7158 0.673 5.5% 5.56 [1.49, 20.80]
Subtotal (95% CI) 39.8% 3.37 [2.25, 5.02]
Heterogeneity: Tau² = 0.00; Chi² = 1.18, df = 5 (P = 0.95); I² = 0%
Test for overall effect: Z = 5.94 (P < 0.00001)
Total (95% CI) 100.0% 2.29 [1.41, 3.72]
Heterogeneity: Tau² = 0.65; Chi² = 69.05, df = 14 (P < 0.00001); I² = 80%
Test for overall effect: Z = 3.36 (P = 0.0008)
Test for subgroup differences: Chi² = 2.25, df = 1 (P = 0.13), I² = 55.5%
0.01 0.1 1 10 100
Favours [high group] Favours [low group]

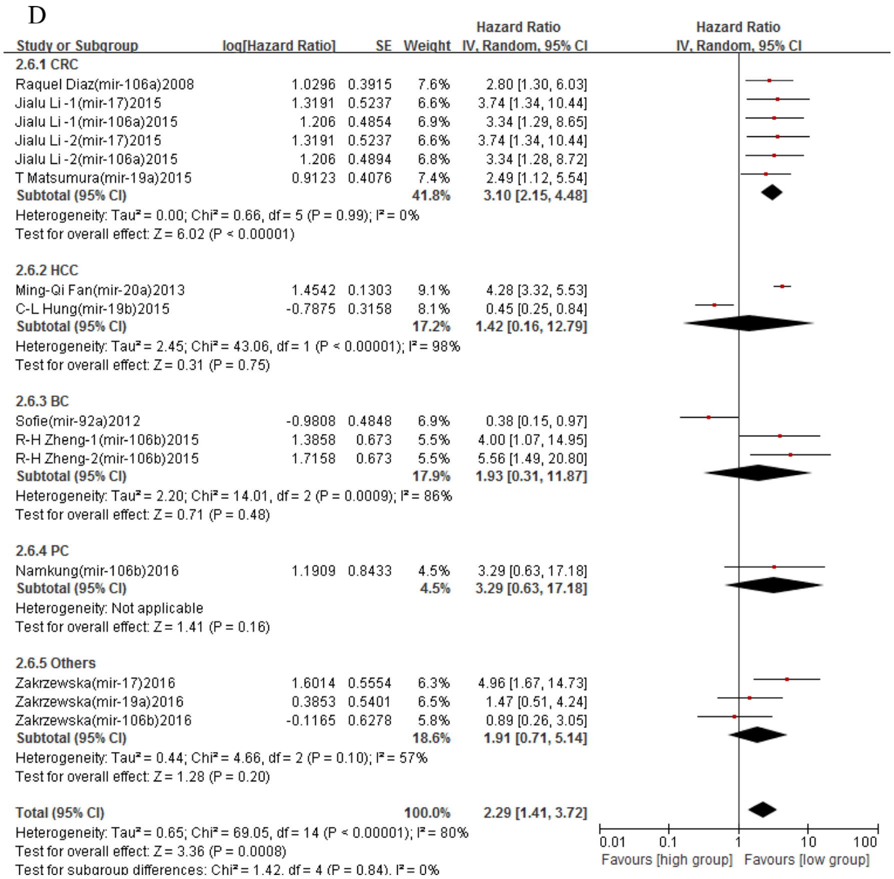

Supplementary Figure 2: (**A**) Forest plot of the association between miR-17-92 family and cancer DFS-unadjusted value. (**B**) Forest plot of the association between miR-17-92 family and cancer DFS-adjusted value subgroup analysis (Asian vs Caucasian). (**C**) Forest plot of the association between miR-17-92 family and cancer DFS-adjusted value subgroup analysis (Tissue sample vs Serum sample). (**D**) Forest plot of the association between miR-17-92 family and cancer DFS-adjusted value subgroup analysis (cancer type).

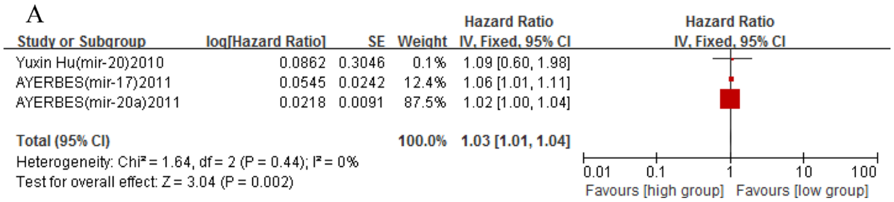

Supplementary Figure 3: (**A**) Forest plot of the association between miR-17-92 family and cancer PFS-unadjusted value.
